# Topoisomerase IIβ targets DNA crossovers formed between distant homologous sites to induce chromatin opening

**DOI:** 10.1038/s41598-020-75004-w

**Published:** 2020-10-29

**Authors:** Mary Miyaji, Ryohei Furuta, Osamu Hosoya, Kuniaki Sano, Norikazu Hara, Ryozo Kuwano, Jiyoung Kang, Masaru Tateno, Kimiko M. Tsutsui, Ken Tsutsui

**Affiliations:** 1 Graduate School of Medicine, Dentistry and Pharmaceutical Sciences, Okayama University, Okayama, Japan; 2 Department of Molecular Genetics, Bioresource Science Branch, Center for Bioresources, Brain Research Institute, Niigata University, Niigata, Japan; 3 Asahigawaso Research Institute, Asahigawaso Medical-Welfare Center, Okayama, Japan; 4 Graduate School of Life Science, University of Hyogo, Kamigori, Hyogo Japan; 5 Institute of Human Complexity and Systems Science, System Science Center for Brain and Cognition, Yonsei University, Seoul, Republic of Korea

**Keywords:** Biochemistry, Molecular biology

## Abstract

Type II DNA topoisomerases (topo II) flip the spatial positions of two DNA duplexes, called G- and T- segments, by a cleavage-passage-resealing mechanism. In living cells, these DNA segments can be derived from distant sites on the same chromosome. Due to lack of proper methodology, however, no direct evidence has been described so far. The beta isoform of topo II (topo IIβ) is essential for transcriptional regulation of genes expressed in the final stage of neuronal differentiation. Here we devise a genome-wide mapping technique (eTIP-seq) for topo IIβ target sites that can measure the genomic distance between G- and T-segments. It revealed that the enzyme operates in two distinctive modes, termed proximal strand passage (PSP) and distal strand passage (DSP). PSP sites are concentrated around transcription start sites, whereas DSP sites are heavily clustered in small number of hotspots. While PSP represent the conventional topo II targets that remove local torsional stresses, DSP sites have not been described previously. Most remarkably, DSP is driven by the pairing between homologous sequences or repeats located in a large distance. A model-building approach suggested that topo IIβ acts on crossovers to unknot the intertwined DSP sites, leading to chromatin decondensation.

## Introduction

Several lines of evidence demonstrated that the beta isoform of type II DNA topoisomerase (topo IIβ), which is reviewed recently^1,2^, is essential for transcriptional regulation of genes expressed in the last stage of neuronal development^3–5^. However, detailed mechanism for this process remains largely unknown. Locating the enzyme’s action sites on the genome would be a logical strategy to elucidate the role of topo IIβ. Type II DNA topoisomerases (topo II) catalyze the interconversion of the spatial positions of two DNA duplexes by a cleavage-passage-resealing mechanism. To discriminate these duplexes, the cleaved (gapped) strand and the other strand that is transferred through the gap are called G-segment and T-segment, respectively^6–8^. Topo II can be cross-linked to the G-segment right at the site of action by treating living cells with etoposide, a topo II-specific ‘poison’-type inhibitor. By taking advantage of this property of the enzyme and immuno-selecting the bound DNA, its action sites on the genome have been mapped in several studies^9–12^. Since the cleaved strand is covalently bound to the enzyme at the 5′ end, this procedure is similar to chromatin immunoprecipitation (ChIP) but essentially different in that no chemical cross-linker is used. Instead, cross-linking is based on the arrested enzymatic reaction intermediate. The procedure thus detects DNA sites directly involved in the reaction and not simply associated with the enzyme.

We have successfully used this type of strategy, termed “etoposide-mediated topoisomerase immunoprecipitation” or eTIP, for mapping of topo IIβ action sites (toposites) in selected genomic regions^11^. In that study we adopted oligonucleotide-tiling arrays to map the bound DNA fragments. In the present study we now extend this technology to a genome-wide scale by massive direct sequencing on NGS (named ‘eTIP-seq’). As a result, mapping resolution was improved significantly and, most importantly, repetitive DNA sequences became a reasonable subject of analysis. Another unique feature of the method is that the immunoprecipitated complex contains not only the covalently linked G-segment but also the T-segment associated with the complex noncovalently, which is released by high-salt treatment and can be analyzed separately afterwards. This provides additional useful information unattainable from conventional mapping techniques.

One of the cellular events where topo II becomes essential is the disentanglement of intertwined chromatid DNAs generated at the final stage of cell division^8,13^. In vertebrates, topo IIα is exclusively responsible for this reaction, namely decatenation. As for topo IIβ, it is believed to relax torsional stresses accumulated locally from various DNA transactions such as transcription^14^. In this reaction called relaxation, the enzyme removes writhes of DNA axis, either positive or negative, by passage between nearby DNA segments. In principle, however, distance between G- and T-segments can be much larger, like hundreds of kilo-bases, although no supporting evidence for this to occur has been reported to date. To detect these distant segmental passage events catalyzed by topo IIβ, we introduced additional steps to eTIP-seq, which include ligation between G- and T-segments via an oligomer adaptor attached to their sheared ends.

Using these techniques, we show here that topo IIβ operates in two distinct modes termed proximal strand passage (PSP) and distal strand passage (DSP) depending on the distance between G- and T-segments. PSP and DSP differ significantly not only in mechanistic sense but also in physiological consequences. While PSP sites are concentrated around transcription start sites (TSS) and also distributes throughout the genome to contribute the relaxation of local torsional stresses, DSP sites are heavily clustered in relatively small number of regions (hotspots) on chromosome. Most remarkable finding is that DSP occurs at the DNA crossovers facilitated by pairing of homologous DNA segments located at a long distance. This is an entirely novel perspective on the cellular function of topo II enzyme, in that a ‘DNA-centric’ mechanism may govern the target selection. The present study suggests that DSP is involved in regulation of higher-order chromatin structures to activate suppressed neuronal genes.

## Results

### Genome-wide identification of topo IIβ target sites by eTIP-seq

**Figure 1:**
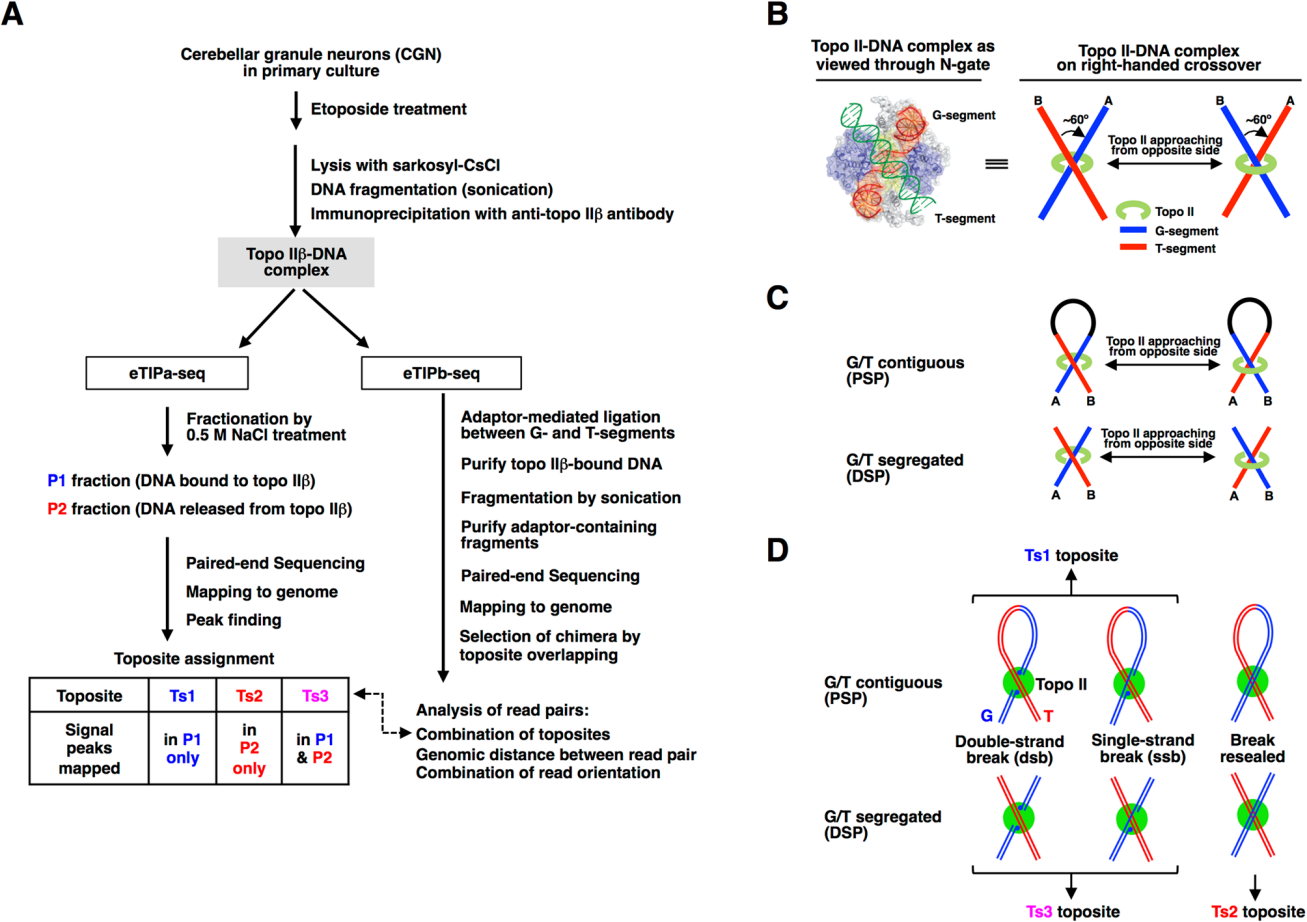
Overview of the eTIP-seq, a mapping technique used in the study and three rules for topo II-DNA interactions that provide the method’s logical basis. (**A**) A flow diagram of eTIP-seq. The topo IIβ-DNA complex in the shaded box was isolated by a procedure similar to eTIP as described previously^11^ (illustrated in Supplementary Fig. S1). DNA in the complex captured on magnetic beads was processed in either way: fractionation by 0.5 M NaCl treatment (eTIPa-seq) or adaptor-mediated ligation (eTIPb-seq). In eTIPa-seq, P1/P2 DNA fractions were analyzed by sequencing and mapped on rat genome. The signal peaks were used to classify the topo IIβ action sites into 3 categories. Experimental results, data processing and toposite assignments for eTIPa-seq are summarized also in Supplementary Fig. S1. The eTIPb-seq procedure is illustrated in detail in Fig. 3A. (**B**) Rule #1. Topo II acts on right-handed DNA crossovers from alternate direction, which selects different duplex as G-segment. The enzyme–DNA configuration depicted in the center is equivalent to the crystallographic representation shown on the left^16^. The two DNA segments at the crossover point are discriminated by ‘A’ and ‘B’. (**C**) Rule #2. G- and T-segments are either contiguous or segregated. As cellular DNA is sheared by sonication before immunoprecipitation, G- and T-segments can reside on different DNA fragments. If the crossover point locates within the same fragment, however, these segments associated with topo II enzyme are contiguous to form a loop. (**D**) Rule #3. The topo IIβ-DNA complex formed under the conditions used for eTIP-seq are constituted of 3 species. Relationships between the cleaved forms and toposites are also indicated in the figure.

The experimental procedure for eTIP-seq analysis is outlined in Fig. 1A. Precursor cells of cerebellar granule neurons (CGN) isolated from infant rats were allowed to differentiate in vitro. Using the same culture system we have shown that topo IIβ is essential for transcriptional induction of a group of genes involved in mature neuronal function^4,11^. In the present study, cells were treated with etoposide at the second day in culture to trap topo IIβ on target DNA. After lysis of treated cells with a detergent sarkosyl and DNA fragmentation, the topo IIβ-DNA complex was recovered on magnetic beads coated with specific antibody to topo IIβ as described previously for eTIP procedure^11^.

The procedure hereafter branches into two routes. In the first route, labeled as eTIPa-seq, the complex bound-DNA on magnetic beads was fractionated into P1 and P2 fractions and sequenced separately on NGS to categorize the topo IIβ target sites termed ‘toposites’. The eTIPb-seq on the right branch determines the G- and T-segments bound to the same topo IIβ molecule by paired-end sequencing of chimeric fragments generated by the adaptor-mediated ligation. To eliminate noise, resulting chimeras were selected by checking whether or not their ends overlap with toposites that are detected in eTIPa-seq.

We first present three fundamental conditions or rules that are critical for the reasoning of eTIP-seq methodology (Fig. 1B-D). Mammalian topo II binds and acts preferentially on DNA crossovers formed between two duplexes. Handedness of the crossover favored by the enzyme is predetermined by structural properties. The enzyme preferentially recognizes DNA crossovers with right handedness, which is energetically favored over left handed ones^15^. In the scheme shown in Fig. 1B left, topo II approaches from the bottom of right-handed DNA crossover, whose wider angle is filled with the enzyme’s identical subunits. Configuration of the two strands illustrated here designates that G-segment is already held by the enzyme and T-segment is approaching to the ‘N-gate’^16^. The same crossover configuration at the ‘DNA gate’ of topo IIβ has been shown recently by a crystallographic study^17^ (they use different handedness definition). In the present study, we adopt this model as a presupposition to interpret the data obtained. It is worth noting that the crossover handedness is the same when the enzyme approaches from either side of the cross but the cleaved strand (G-segment) differs in each case (Fig. 1B center and right). Since the DNA fragments in immuno-selected topo IIβ-DNA complex are sheared by sonication, G- and T-segments can be contiguous or segregated (Fig. 1C). These correspond to the events of proximal strand passage (PSP) and distal strand passage (DSP), respectively (Fig. 3D). The etoposide-induced covalent linkage between topo II and G-segment is reversible under the conditions used for eTIP-seq, in that the enzyme is only partially denatured by sarkosyl and etoposide is diluted out significantly in the process (see Supplementary Fig. S2 for experimental evidence). Because of this, the immuno-captured topo II-DNA complex is composed of 3 species (Fig. 1D). It is also worth noting that the resealed G-segment is still bound on the enzyme and eluted in P2 fraction by 0.5 M NaCl treatment.

### Three classes of topo IIβ target sites (toposites) are identified

**Figure 2:**
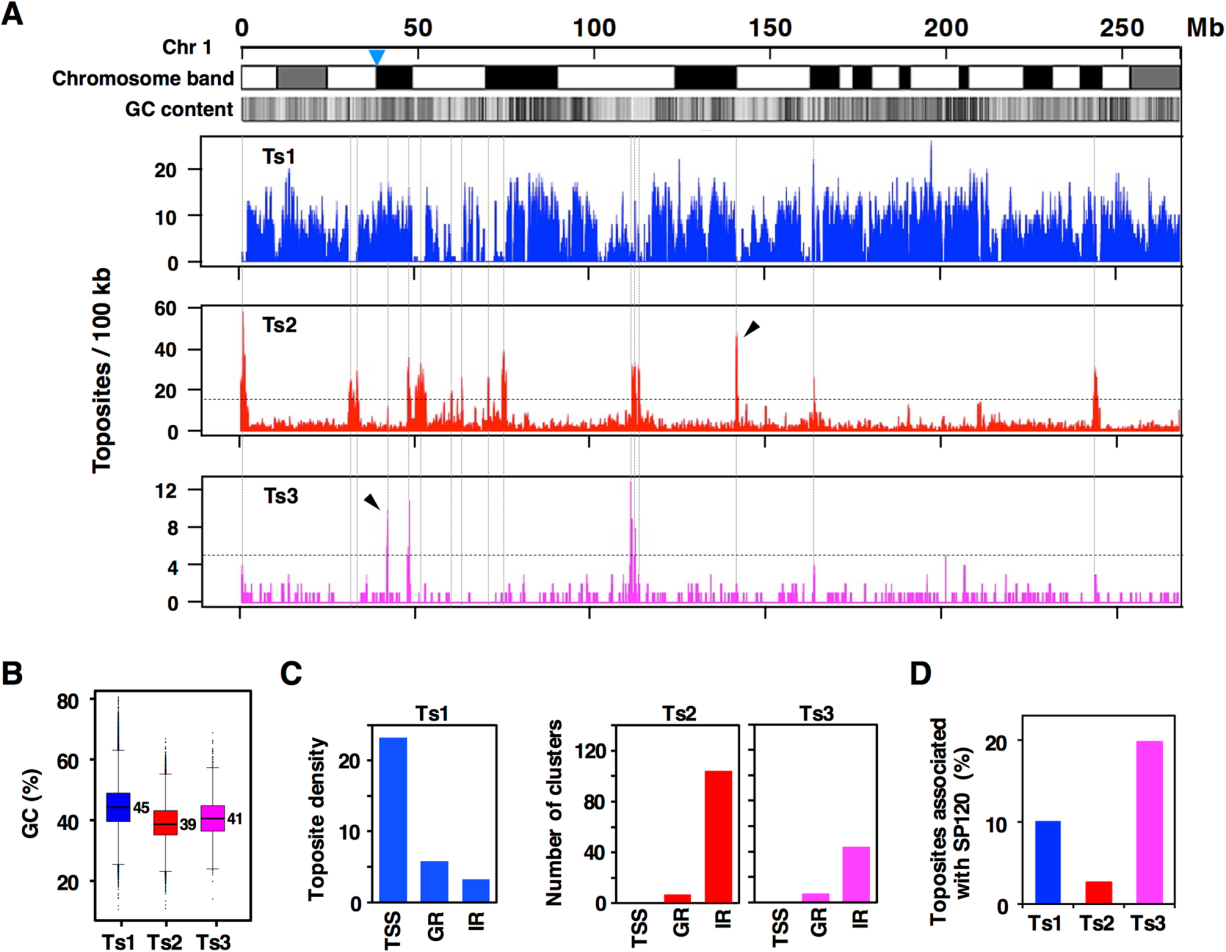
Genomic distribution of toposites and SP120 sites. (**A**) Density map of toposites on entire chromosome 1 as calculated by a sliding window algorithm (100 kb window, 10 kb step). Horizontal dotted lines show thresholds (15 for Ts2, 5 for Ts3) to define high-density peaks that are marked by vertical dotted lines. The position of centromere (p-q boundary) is indicated by nabla symbol and the arrowheads designate typical peaks that clearly distinguish Ts2 and Ts3. (**B**) A box plot of the GC content of toposites. Numbers indicate median values. (**C**) Toposite distribution among the three genomic regions: TSS zone (TSS ± 2 kb), genic region (GR), and intergenic region (IR). TSS zone is excluded from GR and IR. Toposite density is expressed by number of sites per 100 kb, whereas number of clusters is plotted for Ts2 and Ts3 toposites. Definition and positional data for toposite clusters are given in Supplementary Table S2. (**D**) Overlapping situation of toposites with SP120-binding sites (n = 34,300) as determined by ChIP-seq. Site numbers are shown in percentages.

We classified toposites into Ts1, Ts2, and Ts3 by eTIPa-seq (Fig. 1A). The detailed procedure is described in Supplementary Fig. S1. Distribution of toposites in a large scale, a whole chromosome, reveals a strong correlation between toposites and local GC content: Ts1 being located in GC-rich area whereas Ts2 resides in relatively AT-rich area (Fig. 2A). The toposite density plot clearly shows that Ts2 and Ts3 clusters are embedded among Ts1-dense regions in a mutually exclusive manner. Assembly of Ts2/Ts3 in relatively small number of clusters is a feature that distinguishes from Ts1 site. Although Ts2 and Ts3 clusters appear to occupy similar positions in this scale, further analysis demonstrated that Ts2 and Ts3 are distinct toposites with unique characters. As shown later, these clusters constitute hotspots of topo IIβ genomic targets that are likely to be involved in some regulatory processes. Genomic positions of toposites are listed in Supplementary Table S1 and Ts2/Ts3 clusters in Supplementary Table S2. Relative numbers of toposites differ significantly, Ts1 being most prominent (Supplementary Fig. S1).

GC contents of toposites are consistent with the above observation. Ts1 sites are significantly GC-rich as compared to Ts2/Ts3 sites (Fig. 2B). To investigate functional relevance, locations of these toposites were then looked at with respect to characteristic regions: transcription start site (TSS zone), genic region (GR), and intergenic region (IR) (Fig. 2C). Only protein-coding genes are taken into account using the “exRefSeq” which is derived from the rat reference sequence^11^. TSS zone (± 2 kb region encompassing the transcription start site) was included in the analysis as a functional region associated with genes. TSS zone normally overlaps with transcription control region containing promoter and binding sites for transcription factors. Ts1 was highly concentrated in gene-associated regions especially in the TSS zone. In contrast, Ts2/Ts3 sites, as viewed from the number of clusters, were enriched in intergenic regions.

SP120 (hnRNP U/SAF-A/SP120) is a multifunctional nuclear protein that binds both RNA and DNA^18–21^. A recent report shows that the protein binds chromatin-associated RNA and is involved in the organization of chromatin structure in a transcription-dependent manner^22^. Our previous study demonstrated that SP120 is a partner protein of topo IIβ that activates and stabilizes the enzyme through RNA-dependent association^23^. We have also shown that randomly cloned genomic targets of topo IIβ are frequently enriched with SP120-binding sites, suggesting that these proteins bind DNA within close distance^24^. Ts3 toposites frequently overlap with SP120 sites but very little with Ts2 sites, indicating the distinctiveness of these toposites (Fig. 2D).

The SP120 site overlaps also with Ts1 site considerably, implying that at least two distinct genomic sites are involved in the interaction between SP120 and topo IIβ. The eTIPb-seq analysis showed the presence of a looped DNA structure in TSS zone, where Ts1 site is overlapping with SP120 site (Supplementary Fig. S3). Topo IIβ-SP120 complex may somehow be involved in the initiation of transcription.

### The eTIP-seq opens a new perspective on the reaction mode of topo II enzyme

**Figure 3:**
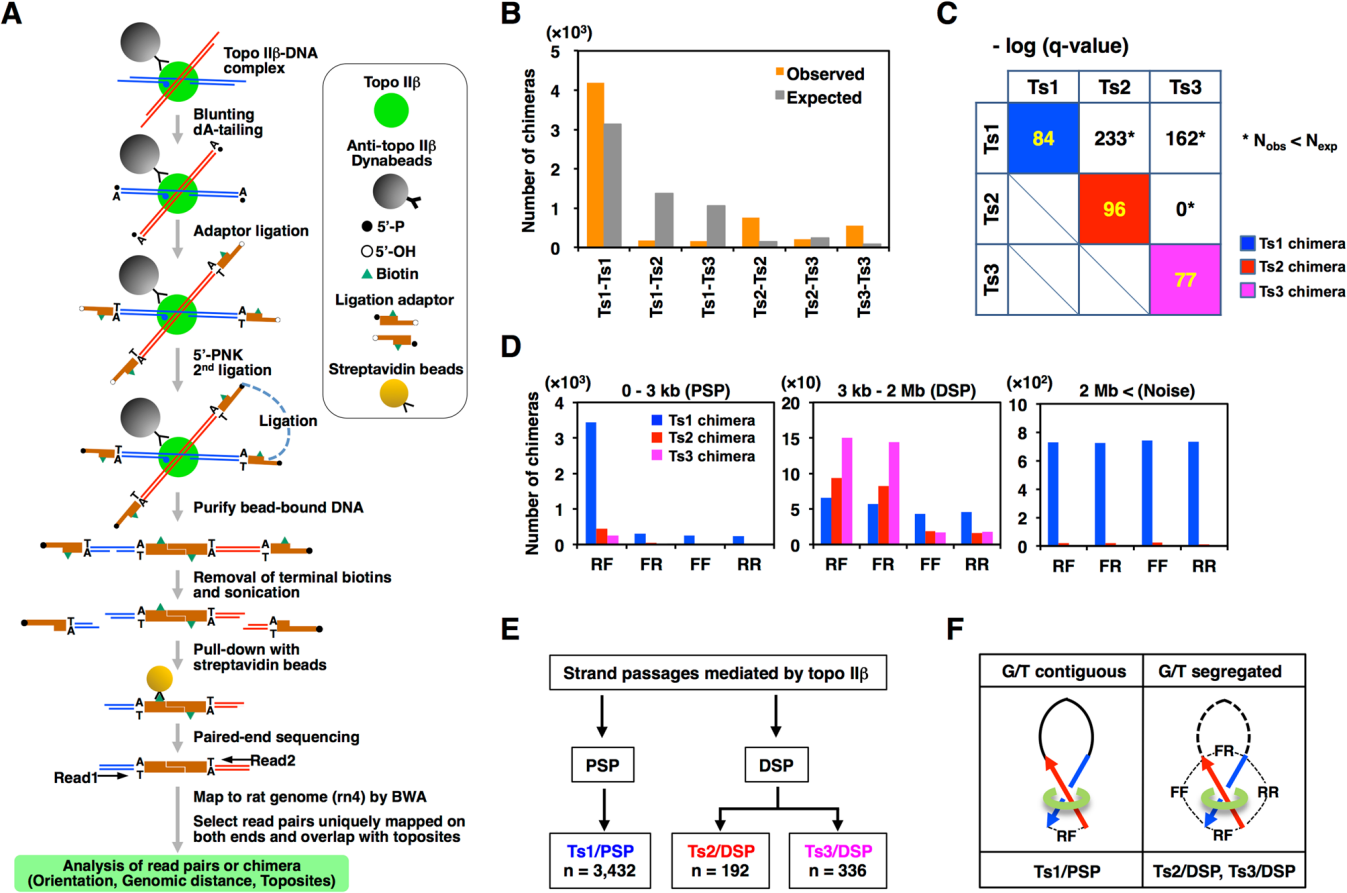
Analysis of chimeras identified by eTIP-seq. (**A**) A flowchart of the eTIPb-seq procedure. The topo IIβ-DNA complex was first immuno-captured on magnetic beads. Ligation reactions were conducted on beads throughout. (**B**) Toposite combinations of intra-chromosomal chimeras. Expected numbers are calculated on the basis of experimental frequencies of toposites detected at the end of chimeras. (**C**) Statistical analysis of toposite combinations. Chi-squared test was conducted between expected and observed frequencies of each combination. Resulting p-values were corrected by Benjamini–Hochberg method to obtain q-values. Shown in the matrix are minus-log transformed q-values. Asterisks indicate the combination where observed number is smaller than expected one. Chimeras with homologous combinations were highly significant and they were referred to as Ts1, Ts2, and Ts3 chimeras. (**D**) Abundance of chimeras in 3 ranges of chimera length (0–3 kb, 3 kb-2 Mb, and 2 Mb <). The first 2 groups correspond to PSP and DSP by definition and long chimeras designated ‘Noise’ are most likely to be due to ‘inter-complex chimeras’ (see text). Ts1, Ts2, and Ts3 chimeras were classified into 4 categories depending on the read combination (RF, FR, FF, and RR), which is designated by read orientations (forward = F, reverse = R) arranged in the order of upstream to downstream on the genome. (**E**) Summary of significant chimeras. Abundance of each chimera group is shown in boxes on the bottom (n). Only Ts1/PSP was considered significant for PSP and Ts1/DSP was regarded as noise signals (see text). (**F**) Probable configuration between topo IIβ and DNA crossovers. Upstream and downstream genomic segments are drawn as red and blue arrows, respectively. Thin dotted lines with read combinations indicate the adaptor ligation between the genomic fragments that are sequenced pairwise from both ends. Thick dotted line on the right represents the lost portion of genomic loops.

To acquire positional information between DNA fragments associated with topo IIβ, we devised a new procedure that includes a ligation step between G- and T-segments via an oligomer adaptor attached to their sheared ends (Fig. 3A). We analyzed the resulting library of paired-read sequences that are referred to as ‘chimeras’. With respect to intra-chromosomal chimeras, various features like chimera length, combination of nearby toposites, read orientations, and genomic locations provide useful information. It should be noted that ‘chimera length’ indicates the map distance between paired reads and not the physical length of chimeric DNA fragments. We first plotted histograms for distribution of chimera length and found that a large proportion of chimera is rather short (51% are shorter than 3 kb). Longer chimeras of certain length, however, do exist and appear as peaks between 3 kb and 2 Mb but chimeras longer than 2 Mb rarely form peaks and their number levels off to a background level. Therefore, only chimeras shorter than 2 Mb will be analyzed hereafter. The data for chimera length distribution are presented elsewhere^38^.

In theory, chimeras must be formed between toposites and can be classified into 6 groups as to the combination of nearby toposites on both ends. From the number of toposites contributing to chimeras, expected number of chimera for randomly chosen toposite pairs of every combination can be calculated. Observed numbers of chimera were then plotted along with expected ones (Fig. 3B). Significance of toposite combinations were determined statistically by comparison of ‘observed’ versus ‘expected’ and the significance levels are summarized (Fig. 3C). The result clearly indicates that only homologous combinations (Ts1-Ts1, Ts2-Ts2, and Ts3-Ts3) are highly favored, whereas heterologous ones are avoided conversely. The presence of heterologous chimeras is probably caused by occasional errors in peak-calling and toposite assignments. Based on these data, only chimeras of homologous combinations will be taken into account and are referred to simply as Ts1, Ts2, and Ts3 chimeras, respectively.

We next examined what sort of information would be available from orientations of read pairs on chimera ends (Fig. 3D). The situation changed dramatically depending on the range of chimera length. In chimeras shorter than 3 kb (PSP, proximal strand passage), RF is by far the most dominant orientation for Ts1, Ts2, and Ts3 chimeras, suggesting that sonication-resistant loop structure (PSP loop) is still present at the ligation step (Fig. 3F). In chimeras between 3 kb and 2 Mb (DSP, distal strand passage), however, the RF dominance in Ts1 chimera is no longer apparent, whereas FR becomes as dominant as RF in Ts2 and Ts3 chimeras. This appears to indicate that the loop structure is disrupted in this range of length and FR is created as an additional combination for ligation (Fig. 3F). As chimera length exceeds 2 Mb, all classes of chimera show no preference in the read orientation, indicating that long chimeras are nonspecific in nature. They are very likely to be originated from the ligation events between neighboring IP complexes fixed on beads, thus termed inter-complex chimera. The same situation can be applied to the Ts1 chimera in the DSP region, which is thus regarded as “noise”. In summary, the Ts1 chimera reflects only PSP events whereas Ts2/Ts3 chimeras are linked to DSP events. The chimera categories and numbers identified are summarized in Fig. 3E. The Ts2/Ts3 chimeras in PSP were ineligible for further analysis. To avoid any confusion on chimera identity, chimeras will be referred hereafter with suffix (/PSP or /DSP) attached to the toposite name.

As this paper specializes only in DSPs, all the results for Ts1/PSP chimeras are presented and analyzed in Supplementary Fig. S3.

### DSP sites are clustered to form a small number of ‘hotspots’

**Figure 4:**
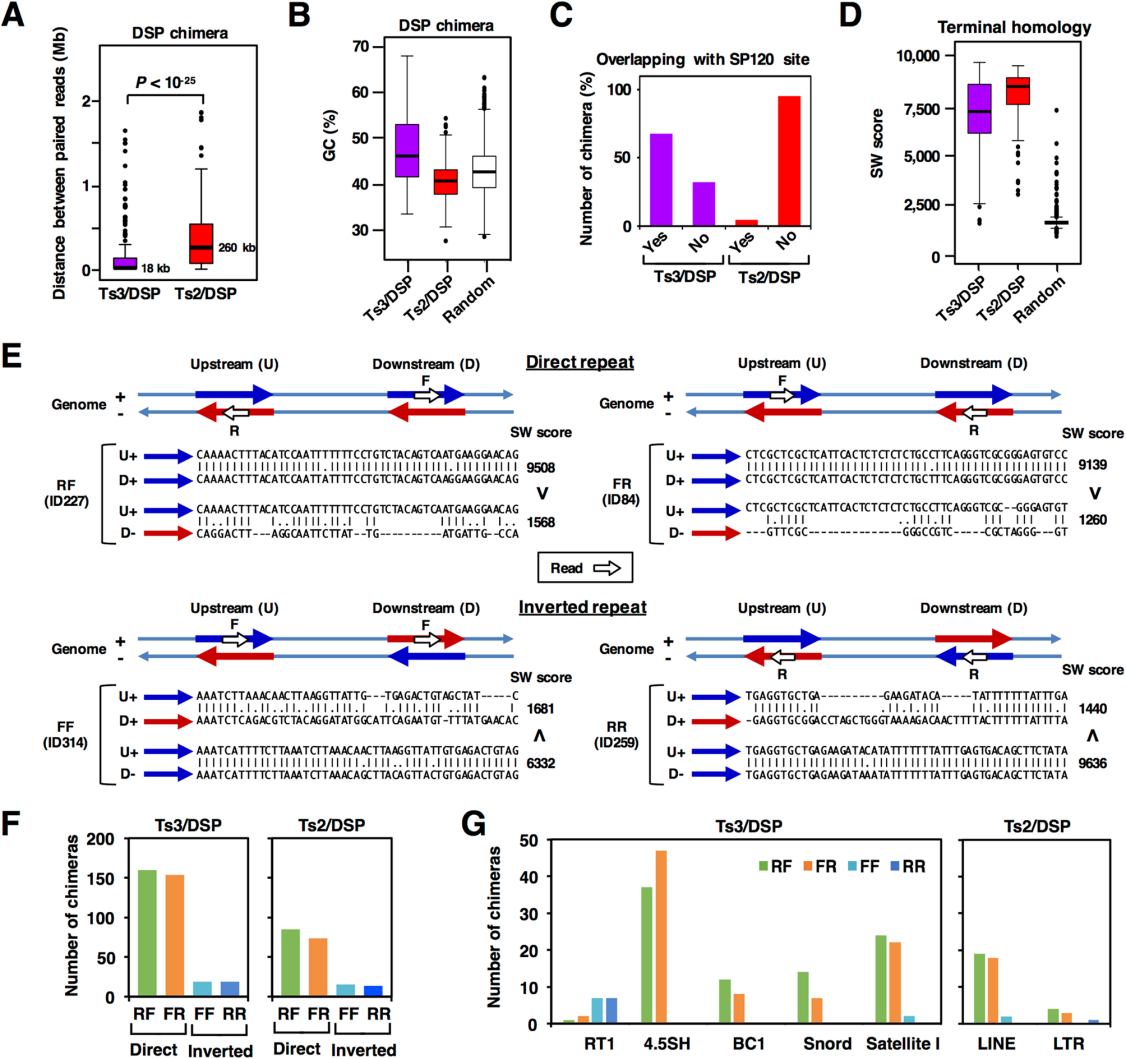
Analysis of DSP chimeras. (**A**) Length distribution of DSP chimeras. Horizontal bars indicate median length. (**B**) GC content distribution of DSP chimeras. As a control chimera, pairing was made between randomly selected 2 kb-segments from Chr 1 that are located within 2 Mb and their gap coverages were less than 20%. (**C**) Coincidence of DSP sites with SP120-binding sites as determined by ChIP-seq. (**D**) Distribution of the SW score for DSP chimeras, which indicates the sequence similarity between the chimera ends. Random pairing was made as in ‘B’. (**E**) Comparison of the combination between read orientations and sequence homology at chimera ends. To exemplify the relationships leading to a general rule, four Ts3/DSPs with different read orientations (RF, FR, FF, RR) were chosen arbitrary from Supplementary Table S5 (specified by their ID in the figure). Two kilobases of genomic segments that contain sequence reads in the center were subjected to homology search by EMBOSS Water program. Homologous segments are referred to as direct or inverted ‘repeat’ depending on their orientation. Pairwise alignments between upstream and downstream segments were done in two ways: plus-strand vs. plus-strand or plus-strand vs. minus-strand (U + vs. D + or U + vs. D-). Aligned sequences in the figure are 50-base region adjacent to the upstream reads. Resulting homology scores (SW score) and inequality signs are indicated on the right. (**F**) Differential abundance of DSP chimeras with direct repeats and inverted repeats at the homologous ends. (**G**) Matching of DSP chimera ends with annotated loci and RepeatMasker entries. RT1 stands for the rat major histocompatibility complex (MHC) genes. 4.5SH, BC1, and Snord are noncoding RNA genes. Satellite I locus resides mostly in pericentromeric regions. Browser view of these regions are presented in Supplementary Fig. S4 and Supplementary Fig. S5.

The length distribution showed that Ts2/DSPs are much longer than Ts3/DSPs (Fig. 4A). Genomic distribution of DSP chimeras showed a great deal of clustering, which often coincided with toposite clusters of the same categories, Ts2 or Ts3. A list of these “chimera-rich regions”, termed DSP clusters, is given in Supplementary Table S3. By comparing the position of these clusters on the genome browser, here we present a new concept termed ‘hotspot’ that integrates the toposite clusters and DSP clusters (Supplementary Table S4). Their relationship is clearly demonstrated by a region in chromosome 1 (Supplementary Fig. S4). Entire hotspots are depicted in a karyogram together with toposite clusters and DSP clusters (Supplementary Fig. S4). With the exception of Chr19, hotspots occur at least once in most chromosomes, indicating that they can be regarded as chromosome landmarks for topo IIβ targets, which is largely limited in number. With reference to gene density profile, the Ts2-hotspot usually resides in gene-poor regions, often adjacent to gene-rich region or “gene city”. This is consistent with the low GC content of Ts2 toposites and Ts2/DSP (Figs. 2B and 4B).

Ts3-hotspots are fewer than Ts2-hotspots and contain several interesting loci of tandemly repeated gene clusters such as noncoding RNA gene clusters and clusters of MHC (major histocompatibility complex) genes. Browser views of representative loci presented in Supplementary Fig. S4 and Supplementary Fig. S5 show that read positions of all the Ts3/DSP chimeras mapped in these regions coincided well with Ts3 toposites and SP120 sites. It is also worth noting that in accord with these sites high FAIRE-seq peaks were detected, indicating that these Ts3/DSP sites stay in an open chromatin conformation all the time in culture. When entire genome was searched, a large proportion of Ts3/DSP overlapped with SP120 sites but Ts2/DSP showed very little overlapping (Fig. 4C).

### Sequences around the DSP chimera ends are homologous each other

In all the featured Ts3-hotspots, repetitive or homologous sequences were present around the chimera ends. To generalize this observation, sequence similarities and read orientations were examined for all Ts3/DSP and Ts2/DSP chimera ends. We do not know the exact position of the crossover targeted by topo IIβ but can assume that it is relatively close to the chimera ends (Fig. 3F). Therefore, from each chimera ends we cut out 2 kb-genomic sequences that contain the reads in the center and measured the homology between the two. Since homologous sequences can reside either on plus (Watson) or on minus (Crick) strand, similarity alignments were made between both strands and the resulting pair of Smith-Waterman scores were compared (Supplementary Table S5). As expected, in all DSP chimeras (both Ts3 and Ts2) either one of the two alignments showed much higher score compared to the other, indicating that chimera ends are indeed highly homologous. This terminal homology in DSP chimeras was significantly higher than control chimera (Fig. 4D). Ts2/DSP showed higher homology than Ts3/DSP.

Since homologous segments of duplex DNA aligned in parallel direction are likely to associate each other^25,26^, this may facilitate the formation of crossover for topo IIβ recognition. Both directions of repeats (direct and inverted) were present in the genome and, most importantly, the combination of read orientation precisely correlated with the directionality of repeats: RF/FR for direct repeat and FF/RR for inverted repeat (Supplementary Table S5 and Fig. 4E). Homologous pairing of direct repeats occurs much more frequently than that of inverted repeats for both DSP chimeras (Fig. 4F). The rule observed here between read orientations eliminates the possibility of inter-complex ligations, in which case the read orientations should be random. Therefore, the overlapping of Ts3/DSP with Ts3 sites shown in Supplementary Fig. S4 implies that the adaptor-mediated ligation in eTIPb-seq protocol indeed occurs between the G-segment and the T-segment bound to the same topo IIβ molecule.

Genome-wide homology search revealed that Ts3/DSP sites are enriched in the loci for MHC genes (RT1), noncoding RNA genes (4.5SH RNA, BC1 RNA, and Snord116) in addition to satellite I (Fig. 4G). The strong bias toward RF/FR orientation indicates that the homologous direct repeats are preferred target of topo IIβ, which was confirmed by examining individual loci for Ts3/DSP (Supplementary Fig. S4 and Fig. S5). The RT1 locus is an exception because FF/RR is a favored combination, which can be explained by the presence of long Ts3/DSP chimeras between the two RT1 gene clusters that originated from a duplication/inverted insertion event, occurred during rodent evolution^27^ (Supplementary Fig. S5). Major repeats like LINE or SINE were clearly minor constituents in Ts3/DSP. In contrast, however, LINE was the prevalent repeat associated with Ts2/DSP (Fig. 4G and Supplementary Fig. S5). It should be noted that a large proportion of chimera ends, 35% for Ts3/DSP and 66% for Ts2/DSP, are not attributable to any known repeats although these end-pairs are also highly homologous each other. We have not pursued their identity any further but they appear to be composed of several sequence groups that tend to cluster in separate DSP hotspots.

### Topo IIβ-dependent alteration of nuclear structure during CGN differentiation

Analyses described hitherto are based on the data from eTIP-seq experiments that are performed at the day 2 of CGN culture, when differentiating cells are most active and abundant^4^. Hereafter, we will analyze the data sets obtained in three conditions: before differentiation at culture day 1 (D1), after differentiation at day 5 in culture (D5), and at day 5 cultured in the presence of ICRF-193, a specific inhibitor of topo II (D5 +). This type of experiment, as described in our previous study^11^, is designed to identify differentiation-dependent changes (D1 vs. D5) and to determine whether or not topo IIβ is required in the process (D5 vs. D5 +).

**Figure 5:**
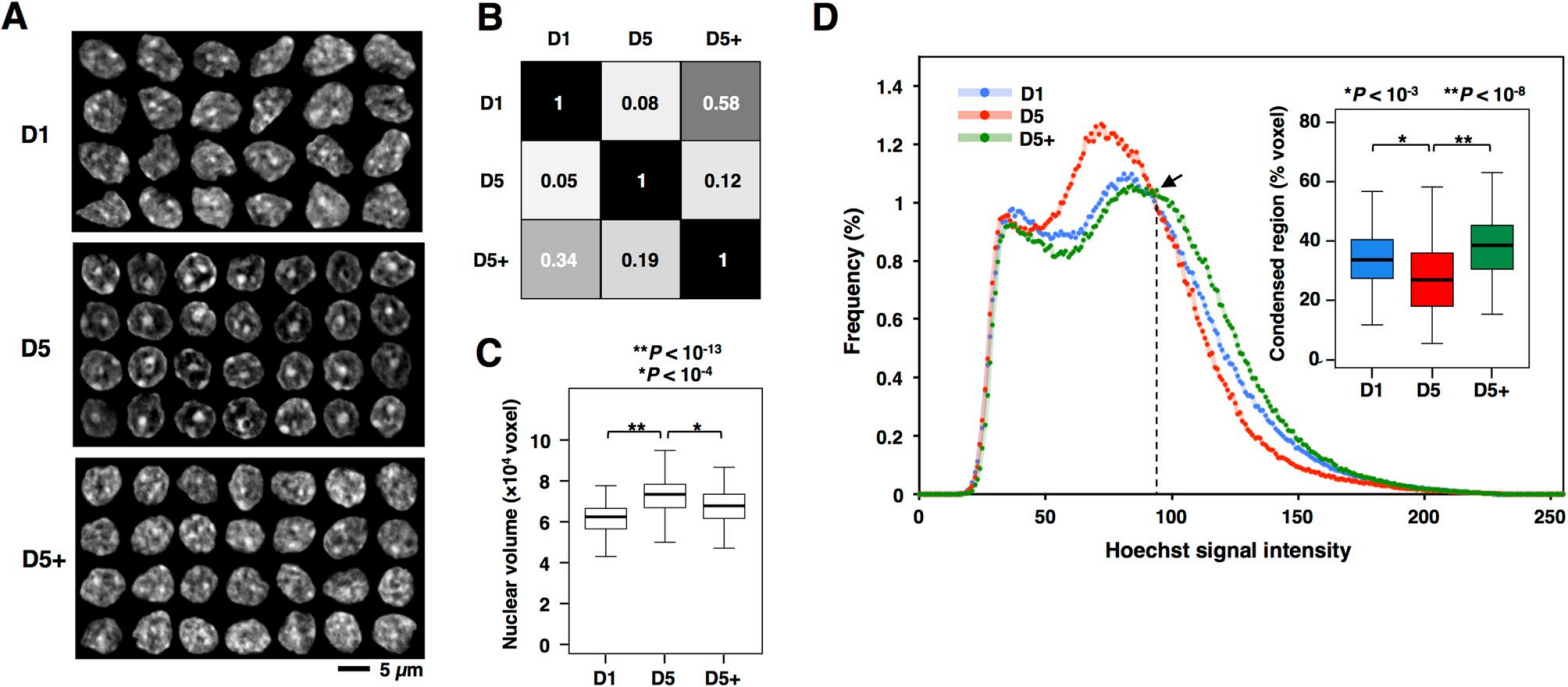
Morphological observations implicating topo IIβ-dependent chromatin opening during CGN differentiation. (**A**) Changes of global chromatin structure. Nuclear DNA in fixed cells was stained with Hoechst 33,342 at the culture days indicated. Nuclear images (z-stack with maximal diameter) were collected and arranged in 3 panels. (**B**) Similarity analysis by a machine-learning program, Wndchrm. Eighty images each was used for learning. Similarity indexes (between 0 and 1) shown are average of 4 trials. (**C**) Measurement of nuclear volume. Horizontal bars in boxplot indicate median values and p-values are from Mann–Whitney U test. (**D**) Degree of chromatin condensation as estimated from DNA staining intensities. The isosbestic point marked by arrow indicates the border between condensed and dispersed chromatin regions. Percentages of condensed region (right side of the curve) were box-plotted (inset).

To assess changes in nuclear shape and chromatin state, cellular DNA was stained with Hoechst. When galleries of nuclear images from D1, D5, and D5 + were compared (Fig. 5A), the nuclear shape after 5 days in culture appeared more round and number of heterochromatic regions that are stained brightly decreased to leave a big blob in the center. However, this change does not occur when topo IIβ was suppressed by ICRF-193 (D5 +), suggesting that the enzyme is involved in the rearrangement of chromatin structure. This result was corroborated more objectively by a machine learning program called Wndchrm for image analysis^28^. The similarity matrix shows that the nuclear appearance is very different between D1 and D5 but it is more similar between D1 and D5 + (Fig. 5B). A 3D image analysis showed that nuclear volume enlarged by 20% during differentiation in a topo IIβ-dependent manner, which probably reflects the chromatin dispersion (Fig. 5C).

To investigate the distribution of nuclear DNA more directly, spectrum of DNA staining intensity was measured (Fig. 5D). The graph represents the histogram of brightness (256 levels in gray scale) obtained from 100 nuclear images for each condition. The three curves had an isosbestic point at the brightness 92–95 (arrow), which was regarded as a boundary between bright (right side) and dark (left side) regions that correspond to condensed (heterochromatic) and dispersed (euchromatic) chromatin compartments, respectively. The ratio of condensed chromatin was calculated from the total voxel number of condensed area and plotted (inset). The result indicates that overall chromatin state shifts toward decondensation in topo IIβ-dependent manner during differentiation.

Taken together, there exists a certain genomic region where repetitive sequences are accumulated as DSP hotspots that serve as a platform for distant chromatin interactions mediated by topo IIβ. Functional consequence of this is likely to be a chromatin decondensation leading to the alteration of transcriptional states of nearby genes (see Discussion).

## Discussion

We have shown in the present study that topo IIβ have two distinctive modes of action, PSP and DSP, on genomic DNA. DSP is a novel mode of action in that DNA crossovers targeted by the enzyme are likely to be triggered by association of homologous DNA segments located at a distance.

The homology-sensing can occur between chromatin segments, not necessarily between naked DNA, because oligo-nucleosomes with identical DNA sequence were shown to associate selectively in solution^29^. Pairing of homologous sequences is an essential step for important biological processes such as meiotic pairing of homologous chromosomes. However, little evidence has been published on the physical basis for the association between duplexes with homologous sequences. Only recently, some theoretical approaches suggested that interaction between the two duplexes of DNA that are aligned side-by-side is most stable if the pair has a certain sequence homology and the direction of alignment is parallel instead of anti-parallel^25,30^. This mechanism may be adopted to explain our results indicating the terminal homology of DSP chimeras (Fig. 4D).

**Figure 6:**
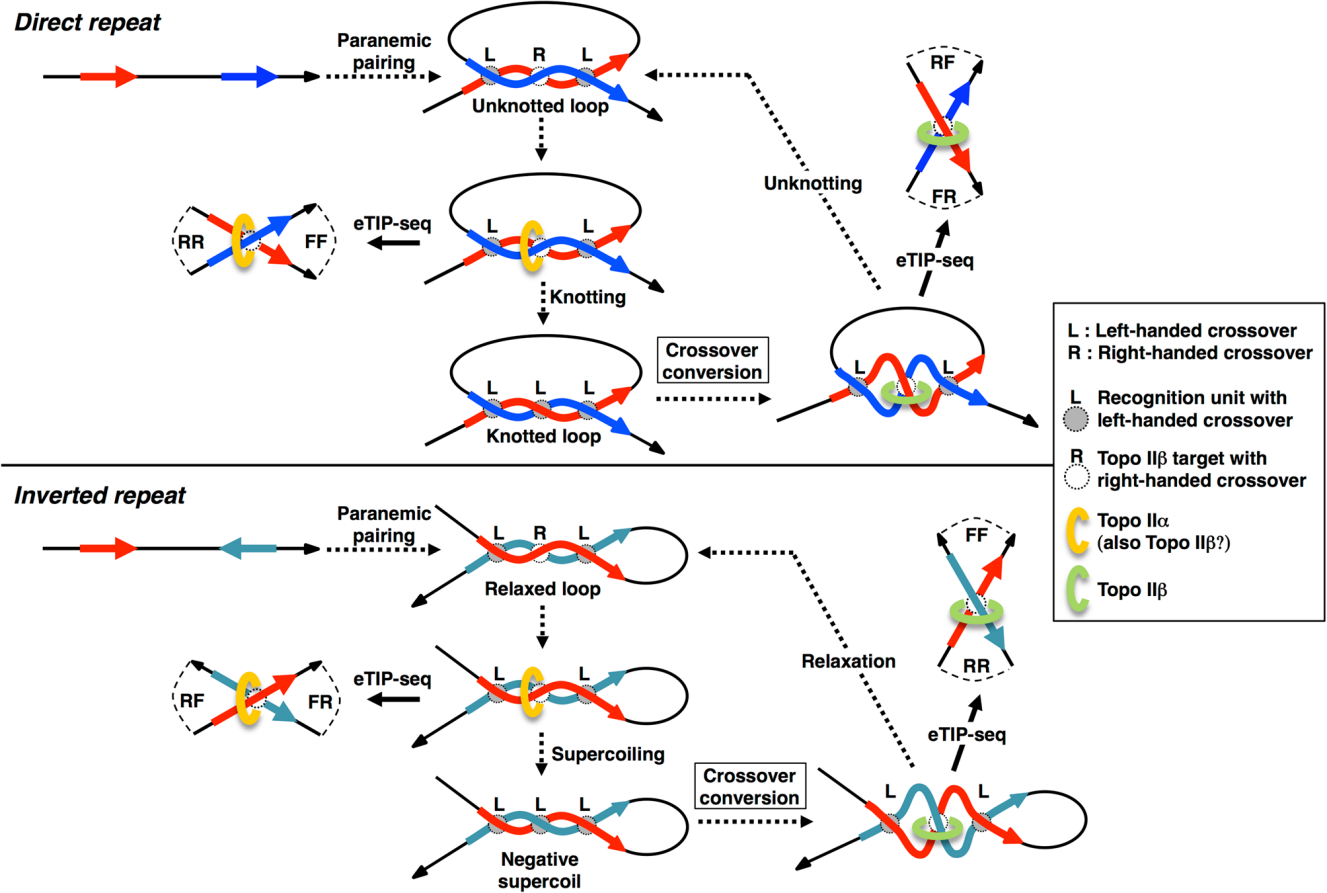
Homologous pairing between repetitive DNA segments at the DSP site: a model. Topo II action at these sites leads to different consequences depending on the repeat orientations, direct and inverted. Plus-strand DNA path from 5′ to 3′ direction is depicted by black arrows. Homologous segments are shown by red arrows (upstream) and blue arrows (downstream) on plus strand or by dark-green arrows on minus strand. Homologous pairing between duplex DNAs aligned in parallel starts by ‘paranemic’ mode. Left-handed crossovers facilitate the interaction between major grooves to form ‘recognition unit’, which is a quadruplex structure required for stable pairing^25^. Since the pairing occurs only when the two DNA segments are aligned in parallel, direct and inverted repeats bring about very different results both in the loop configuration between paired repeats and in the topological structure generated after topo II action. Direct and inverted repeats result in knotted loops and negative supercoils, respectively. The ‘crossover conversion’, which is an energetically favored step, is a mandatory process for the reverse reaction (unknotting or relaxation) to occur. The eTIP-seq experiments performed in the present study produced dominant DSP chimeras with RF/FR read orientations that were originated from direct repeats. This suggests that in terminally differentiating CGN cells topo IIβ is almost exclusively involved in unknotting reactions (illustrated in the upper right).

Here we propose a model that implies the presence of two distinct topo II-involved processes: formation and disruption of higher order chromosomal structures (Fig. 6). Initial pairing is likely to occur in ‘paranemic’ mode^25^. Since major grooves of paired duplex face each other at the left-handed crossover^16^, additional hydrogen bonds can be formed between identical base pairs in the paired DNA as suggested to occur in four-stranded DNA structure^25,26^. Using computational methods, Mazur proposed a convincing model for the mutual recognition between two homologous duplex DNAs through direct complementary hydrogen bonding of major groove surfaces in parallel alignment^25^. At the contact area, a quadruplex structure is formed between the duplexes associated with left-handed crossover configuration. The quadruplex can extend three to four consecutive base pairs called ‘recognition unit’ that have to be spaced by at least several helical turns. Therefore, the pairing requires relatively long stretches of DNA, but only partial homology. A right-handed crossover is generated between the recognition units, which is a preferred target of topo II. After strand passage between the direct repeat (homologous pairs are on the same strand), number of recognition units are doubled and the pairing mode turns to intertwined mode introducing knots into the looped domain between the homologous pairs (Fig. 6, upper section). In contrast, when homologous pairs are on opposite strands (inverted repeat), negative supercoils, instead of knots, are produced within the looped domain (Fig. 6, lower section).

The paranemic association between homologous pairs should be quite unstable without the consecutive action of topo II. Although, in principle, the topo II enzyme involved here can be either topo IIα or topo IIβ, topo IIα is the feasible one since it is the major enzyme in cycling cells that is required for the mitotic chromosome condensation in G2/M phase^31^. We speculate that the knotting reaction between LINEs, an abundant repetitive element in mammals, could significantly contribute to the chromatin condensation along the chromosome axis. Roles of repetitive elements in organization of higher-order chromatin structures have been proposed^32^.

The model predicts that at the time of eTIP-seq analysis (day 2 in CGN culture) topo II is engaged in the removal of knots between direct repeats or removal of supercoils between inverted repeats because the combination of read orientations detected in DSP chimeras was exclusively RF/FR for direct repeats and FF/RR for inverted repeats (Supplementary Table S5). The left-handed crossover in the knot will be converted to right-handed configuration by thermal wobbling before the action of topo IIβ^15,16^. We can state definitively that topo IIβ is the enzyme involved in these reactions since β-specific antibody was used for eTIP-seq and topo IIα is not expressed in differentiating neuronal cells. We emphasize that eTIP-seq can discriminate the directionality of topo II reaction, knotting or unknotting, by determining the read orientations on the chimera between direct repeats. The favored read orientations, RF/FR over FF/RR, in DSP chimeras with direct repeats suggests that unknotting is the preferred reaction of topo IIβ probably because knots are detrimental to transcription^33,34^. The unknotting between distant chromatin sites would certainly lead to an opening or decondensation of global chromatin structures as observed in this study (Fig. 5). The reason why inverted repeats are only minor target of the enzyme would be that negative supercoils are constantly removed by the action of topoisomerase I. There might be some protein factors involved in additional stabilization of DNA crossovers at DSP sites. Since about 68% of Ts3/DSP is overlapped with SP120 sites (Fig. 4C), the protein may serve as a crossover stabilizer at these sites. But it is not so at Ts2/DSP sites where SP120 is almost absent. Although the possibility that other protein is responsible for the crossover stability cannot be eliminated, we suggest that homologous pairing at Ts2/DSP sites can be conducted purely by ‘DNA-centric’ manner without any assistance from proteins.

To date, transcriptional regulation by topo IIβ has been attributed to its action within relatively narrow DNA regions like promoters or enhancers^35,36^. Related observation in the present study would be that Ts1 toposites are highly enriched in TSS zone (Fig. 2C). When compared to other mapping technique for topo IIβ-generated DSBs (END-seq)^10^, about 46% of END-seq peaks in mouse cortical neurons overlapped with Ts1 toposites (see Supplementary). Furthermore, the pattern of Ts1 distribution around TSS (shown in Supplementary Fig. S3) was very similar to that of DSB detected by END-seq in human cells^37^.

It is unable to exclude the possibility that the observed inhibition of chromatin de-compaction by ICRF-193 (Fig. 5) is partly caused by the inhibition of topo IIβ-dependent removal of the torsional stress in highly expressed genes. However, we suggested by mRNA-seq analysis that the alteration of chromatin compaction may not be explained solely by the effects of inhibitor on transcription in terminally differentiating neurons (see Supplementary). As to the involvement of topo IIβ in ongoing transcription, the present study implicated that the relaxation of transcription-generated supercoils occurs at the Ts1/PSP sites within genic regions (Supplementary Fig. S3). In contrast, Ts2/DSP and Ts3/DSP sites are likely to be involved in the de-compaction of global chromatin structure during differentiation that is inhibited by ICRF-193 (Fig. 5). More complete analyses on the relationship between chromatin higher-order structures and gene expression are described elsewhere^38^.

## Methods

### Animals

All animal care and experimental details have been approved by the Animal Experiment Committee of Okayama University (authorization number: OKU-2012108, OKU-2014084, and OKU-2018265). All animal experiments were conducted in accordance with the Guidelines specified by the Regulation for Animal Experiments of Okayama University.

### Primary culture

The primary culture was started with cerebellar tissue obtained from Wistar rats of 8 days after birth as described previously^11^. Cells maintained in culture are mostly postmitotic cerebellar granule neurons (CGN) that continue to differentiate in vitro. When it is required, topo IIβ activity was inhibited specifically throughout the culture period by daily addition of 10 µM ICRF-193 that also degrades the enzyme^39^ (first addition was made at 12 h). Cells were harvested either at day1 (D1), day 2 (D2), day 5 (D5) or day 5 in the presence of ICRF-193 (D5 +) depending on the experiment.

### eTIPa-seq

Samples were prepared by modifying the protocol described previously^11^. CGN cells cultured in 100-mm dishes for 2 days were treated with 0.5 mM etoposide (VP-16) in serum-free medium for 15 min. The cells on dish were lysed with 1% sarkosyl and the lysate was passed through a 23 G needle. After adding concentrated CsCl to a final concentration of 0.5 M, the mixture was sonicated with a microprobe, and diluted with 3 volumes of TEST-100 buffer (10 mM Tris–HCl: pH 7.5, 10 mM EDTA, 100 mM NaCl, 0.1% Triton X-100, and protease inhibitors). Topo IIβ cleaved complex in the supernatant was immuno-captured overnight at 4 °C with Dynabeads Protein G coated with a specific monoclonal antibody to topo IIβ (3B6)^4^. Beads were washed three times with TEST-200 (the same as TEST-100 except that NaCl concentration is 200 mM).

Washed beads were treated with TEST-500 buffer (the same as TEST-100 except that NaCl concentration is 500 mM) at 4 °C to elute the noncovalently associated DNA (P2 fraction) from the bead-bound DNA (P1 fraction). After digesting with RNase A and proteinase K, DNA was purified by phenol/chloroform extraction followed by ethanol-precipitation with carrier glycogen.

Purified DNA fractions (P1 and P2) were sheared to ~ 200 bp using Covaris S2 sonicator. The eTIPa-seq libraries for NGS sequencing were prepared using the TruSeq DNA LT Sample Preparation Kit (Illumina) according to manufacturer’s instructions. After 10 cycles of PCR amplification, quality of the library was monitored by Agilent 2100 BioAnalyzer. Paired end sequencing (100 bp each) was performed on Illumina HiSeq 2000.

### eTIPb-seq

Entire procedure is illustrated schematically in Fig. 3A. The immuno-captured topo IIβ-DNA complex (IP complex) bound to magnetic beads was prepared as in the eTIPa-seq procedure. After washing with TEST-200 buffer, sheared DNA ends were blunted by T4 DNA polymerase and dA-tailing of 3′ ends was done with NEBNext module mix (Klenow fragment, proteinase inhibitors in NEB reaction buffer). To attach the adaptor to the ends of topo IIβ-associated DNA fragments, beads were incubated for 6 h at 22 °C in adaptor ligation mix (12,000 units of T4 DNA ligase, 5% PEG-8000, 1.5 µM ligation adaptor DNA, proteinase inhibitors in NEB ligation buffer) and the 5′ end of ligation adaptor was phosphorylated by T4 polynucleotide kinase. The ligation between adaptors (2^nd^ ligation) by T4 DNA ligase was performed over night at 16 °C with continuous rotation. The reaction was terminated on ice with 10 mM EDTA and the bead-bound DNA (eTIPb-seq library) was purified as in eTIPa-seq procedure. The ligation adaptor DNA was prepared by annealing complementary oligomer DNAs (Operon): 5′-GTTGGATCCGATA[Bio-dT]CGC-3′ (top strand) and 5′-GGCCGCGATATCGGATCCAACT-3′ (bottom strand). Top strand contains a biotinylated deoxy thymidine (Bio-dT) and bottom strand has 3′-dT tail and 5′ overhang of GGCC for the 2nd ligation between adaptors. To anneal the oligomers, equimolar amounts of oligos were placed in boiling water for 5 min and then allowed to cool slowly to room temperature. The completion of annealing was confirmed by polyacrylamide gel electrophoresis.

To remove the biotinylated nucleotide from terminal adaptors, the eTIPb-seq library DNA was incubated with T4 DNA polymerase in the presence of dATP and dTTP. Purified DNA was sheared to ~ 200 bp by Covaris sonicator. The biotinylated DNA fragments were captured with Dynabeads MyOne Streptavidin C1 (LifeTechnologies) and the beads were washed 3 times with Tween wash buffer. The fragmented DNA on beads was end-repaired, dA-tailed and ligated with paired-end sequencing adaptor, followed by PCR amplification (15 cycles with PCR primer cocktail and PCR master mix, Illumina). Amplification products were purified using AMPure XP beads (Beckman) according to manufacturer’s instructions. Quality of the resulting library was monitored using Agilent 2100 BioAnalyzer. Paired end sequencing (100 bp each) was performed on Illumina HiSeq 2000.

### ChIP-seq

A standard procedure was used for SP120 ChIP. Briefly, the CGN cells at the 2nd day in vitro (D2) were fixed with 1% formaldehyde for 10 min and quenched with 125 mM glycine for 5 min at 25˚C. Washed cells were scraped off, collected by centrifugation and stored at − 80 °C until use. The frozen cell pellets were resuspended in SDS-lysis buffer containing 0.8% SDS and incubated on ice for 10 min. The cell suspension was diluted with 3 volumes of the lysis buffer without SDS (ChIP dilution buffer) and sonicated for 2 min with intermittent poses by ultrasonic disruptor (UD-201, TOMY) attached with a special microtip (power setting at 5). After twofold dilution with ChIP dilution buffer, pre-clearing with normal mouse IgG, the final lysate was used for immunoprecipitation with anti-SP120 monoclonal antibody (1-67D^23^) bound to Dynabeads Protein G. The beads were washed consecutively with RIPA buffer, 500 mM-NaCl RIPA buffer, LiCl buffer, and 0.1% Triton in 10 mM Tris–HCl (pH8). Elution was performed twice with an elution buffer containing 1% SDS. After adding 5 M NaCl to a final concentration of 0.2 M, the combined eluate was heated at 65 °C for 4 h to reverse the cross-link. Sixty nanograms of purified DNA was subjected to size selection (100–600 bp range) by AMPure XP beads (Beckman) and fragmented by Covaris to generate ~ 200 bp segments. The fragmented DNA was end repaired, dA-tailed and ligated with paired-end sequencing adapter. Purified DNA was amplified with 14 cycles of PCR using PE PCR 1.0 and PE PCR 2.0 primers (Illumina) and the libraries were paired-end sequenced (100 bp each) on Illumina MiSeq platforms.

### FAIRE-seq

We used the FAIRE-seq technique to map the genomic regions that are devoid of associated proteins and thus accessible to protein factors including topo II. Except for some modifications, we followed the experimental procedures for FAIRE (formaldehyde-assisted isolation of regulatory elements) described previously^40^. NGS protocols were the same as used for eTIPa-seq.

### Mapping and peak-calling of sequence data

For eTIPa-seq and ChIP-seq analyses, the FASTQ paired-end read data were mapped by BWA against rat reference genome (UCSC rn4) and then filtered to remove frequently encountered experimental artifacts. Only paired reads of 100 bp with correct orientation and reasonable distance (< 500 bp) were selected. To eliminate repetitive sequences that are mapped multiply on genome, only uniquely mapped pairs on both ends were used for peak calling. R-package ZINBA version 2.02.03 was used to find peaks by setting ‘refinepeaks’ to 1, ‘threshold’ to 0.5, ‘extension’ to 200 and other factors to default^41^. Selected fragments that are flanked by uniquely and correctly mapped read pairs were used to construct wiggle track formats. Pileup data were calculated by ‘basealigncount’ function of ZINBA with extension of 200.

For eTIPb-seq analysis, the FASTQ paired-end read data were mapped by BWA to rn4 separately as ‘read 1′ and ‘read 2′. Sequence reads with the ligation adaptor sequence at 5′ end were discarded. Only uniquely mapped pairs were used for further analyses.

For FAIRE-seq analysis, the wiggle data were formatted through the same procedure as used in eTIPa-seq.

### Microscopy and image processing

CGN cells at day1 (D1), day 2 (D2), day 5 (D5) or day 5 in the presence of ICRF-193 (D5 +) were fixed with paraformaldehyde and stained with Hoechst 33,342. Fluorescence images were acquired by using AxioVision 4.5 software through AxioCam MRm camera installed on an Axiovert 200 M inverted fluorescence microscope with an ApoTome optical sectioning system (Carl Zeiss).

Serial 25 optical sections were collected from 100 nuclei at 0.275 µm z-axis intervals. Measurements were performed by ImageJ software. The z-stack was cropped to obtain regions of interest. The cropped z-stack was then converted to 8-bit gray scale and subjected to gray-level histogram quantification as follows. The stacks were normalized by linearly stretching the voxel intensities over a full range of 256 Gy-levels with the ‘Histogram’ tool (termed normalized-stacks) and smoothed by a mean filter (filtered-stacks). Using the normalized-stacks, three-dimensional nucleus segmentation was automatically performed by the aid of the NucleusJ plugin ‘Nucleus segmentation’ function^42^ (segmented-stacks). The corresponding filtered- and segmented-stacks were combined by ‘Subtract in the Image calculator’ (combined-stacks). By using the ‘Histogram’ tool, intensities of all voxels within the combined-stacks were computed, expressed as histogram plots, and exported as text files.

To estimate the degree of chromatin condensation at various culture days, the staining intensity for 100 nuclei was recorded (8 bit, 256 Gy scale). After normalizing the intensity to total intensity per nucleus, intensity versus median frequency curve was plotted. The isosbestic point for D1, D5, and D5 + curves indicates the border between condensed and dispersed chromatin regions. The condensed region corresponds to the right side of isosbestic point.

### Statistical analysis

For the continuous variables of interest, the Mann–Whitney U test with two-sided significance (wilcox.exact function of R-package, exactRankTests) was used if the overall difference was statistically significant. Box plots were drawn by R with default settings. The chi-square test or Fisher’s exact test (chisq.test or fisher.test function of R) was used to examine the differences in the categorical characteristics and changes in the groups. Analyses with R-package were conducted according to R Core Team, R: A language and environment for statistical computing, R Foundation for Statistical Computing, Vienna, Austria (2018), available online at https://www.R-project.org/.

## Supplementary information

Supplementary Table S1.

Supplementary Table S2.

Supplementary Table S3.

Supplementary Table S4.

Supplementary Table S5.

Supplementary Information.

## Data Availability

The raw data obtained in this study are available from DDBJ Read Archive (https://ddbj.nig.ac.jp//DRASearch/) under accession numbers of DRA007399 for eTIPa-seq, DRA007479 for eTIPb-seq, DRA007375 for SP120 ChIP-seq, DRA002525 for mRNA-seq and DRA007436 for FAIRE-seq.
