# Supplementary material for: Topoisomerase IIβ targets DNA crossovers formed between distant homologous sites to induce chromatin opening
Source: Sci Rep. 2020 Oct 29;10:18550. doi: 10.1038/s41598-020-75004-w (PMC7596052; doi:10.1038/s41598-020-75004-w)
Supplement: Supplementary file 6 — Supplementary Information. [file 41598_2020_75004_MOESM6_ESM.pdf]

Topoisomerase IIβ targets DNA crossovers formed between distant homologous sites to induce chromatin opening

Mary Miyaji, Ryohei Furuta, Osamu Hosoya, Kuniaki Sano, Norikazu Hara, Ryozo Kuwano, Jiyoung Kang, Masaru Tateno, Kimiko M. Tsutsui and Ken Tsutsui

1. Notes on methodology

This section contains information on fundamental principles and experimental evidence of eTIPa-seq.

Classification of topo IIβ target sites (eTIPa-seq)

Treatment of the immunoprecipitate with 0.5 M NaCl separates the DNA into P1 and P2 fractions (Fig. S1A, Step 5). P1 is mostly composed of DNA fragments covalently bound to the enzyme whereas the association of fragments released in P2 is noncovalent in nature. Note that resealed G-segment is generated after lysis with sarkosyl-CsCl (step 2) but still associated with the enzyme during the following steps (steps 3 and 4), and recovered in P2 fraction after high-salt treatment (step 5). This behavior of G-segment will be explained by a model experiment shown later.

A large proportion of recovered DNA fragments distributed between 0.5 kb and 3.0 kb under these conditions (Fig. S1B). Upper limit of the smear (~3 kb) reflects the maximal size of sonication-resistant DNA, which was constant between experiments. For the maximal mapping resolution, shorter fragment size would be desirable here but we avoided stronger shearing conditions to retain noncovalently bound fragments, whose presence is essential for eTIPa-seq. Densitometric scanning of the smear showed that the fragment size peaks around 1.5 kb (Fig. S1C). Relative DNA yields from both fractions showed a strong dependency on etoposide treatment (Fig. S1D). Purified DNA from these fractions was subjected to sequencing on NGS. Sequence reads from eTIPa-seq experiments were processed as in Fig. S1E. Paired-end reads for the DNA fragments purified from P1 and P2 fractions were mapped on the rat reference genome, UCSC rn4 (Baylor Build 3.4, November 2004). We have adopted this version because more recent versions (rn5 and rn6) contained multiple rearrangements probably due to erroneous fragment assemblies. The mapped fragments between the read pairs were analyzed by a peak-finding program called ZINBA[1]. We defined 3 distinctive categories of topo IIβ-targeted sequences called toposites (Ts1, Ts2, and Ts3) by using ZINBA peaks for P1, P2, and their overlaps (Fig. S1E, bottom). In summary, toposites are regions with P1 peak alone (Ts1), P2 peak alone (Ts2), and both P1/P2 peaks (Ts3).

Genomic position and site length of resulting toposites are listed in Table S1.

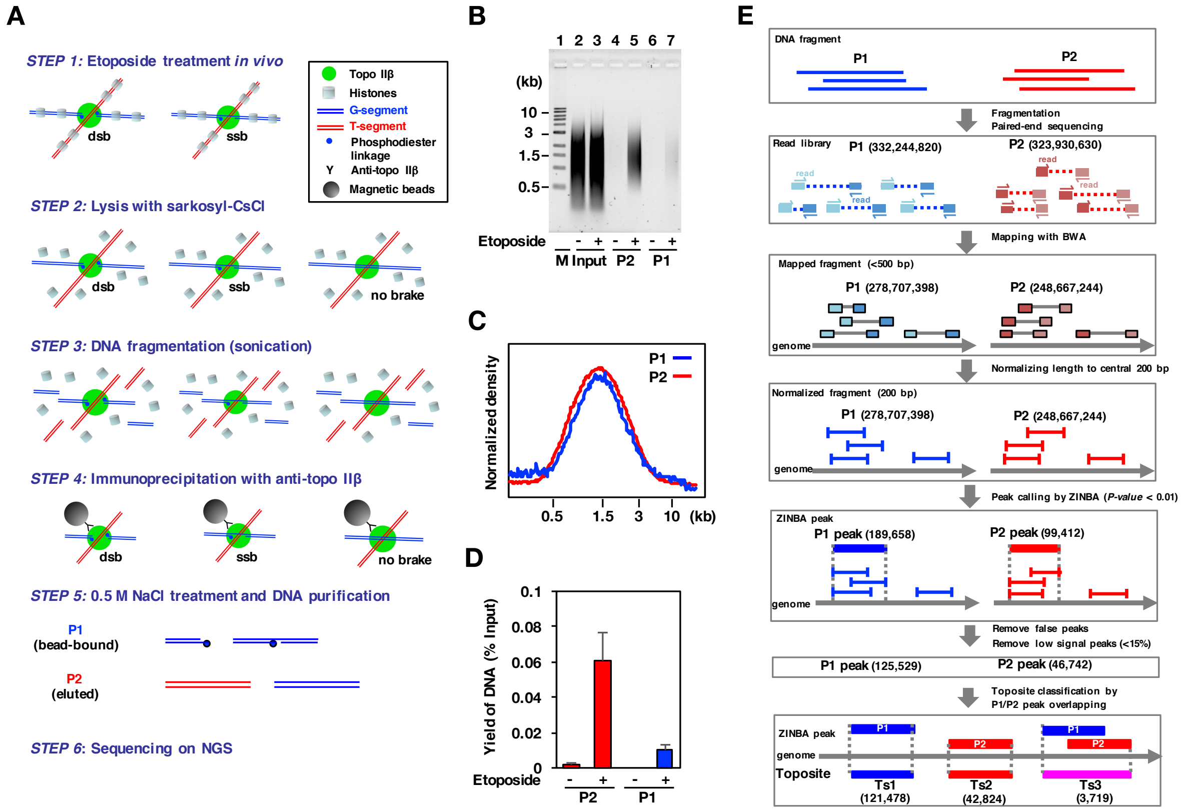

Supplementary Fig. S1 Partitioning of DNA fragments among fractions, and toposite assignments in eTIPa-seq. (**A**) Schematic representation of the experimental procedure shown in Fig. 1A. Dsb, double-strand breakage; ssb, single-strand breakage. (**B**) The fragment size of DNA from P1/P2 fractions as determined by agarose gel electrophoresis. The same procedure was conducted in the presence (+) or absence (-) of etoposide. M, size marker; Input, sonicated DNA before immunoprecipitation. (**C**) Fragment size distribution of P1/P2 fractions from etoposide-treated cells. There was no difference between the normalized densitometric profiles of DNA from P1/P2 fractions. (**D**) Relative amounts of DNA recovered from P1/P2 fractions. Concentration of purified DNA was measured by Quant-it PicoGreen dsDNA kit (Invitrogen). (**E**) Schematic representation of the entire signal processing. DNA amounts used for sequencing were 485 ng for both P1 and P2 fractions. Numbers in parentheses indicate actual signal numbers selected in each step. For P1/P2 overlaps, the sum area is regarded as a Ts3 peak if more than half portion of the shorter peak region is overlapped. P1 and P2 peaks showed little self-overlapping.

Most significant factor in eTIPa-seq is that the etoposide-induced covalent linkage between topo IIβ and G-segment is partially lost by breakage-resealing activity of the enzyme

treated with sarkosyl-CsCl. To obtain experimental evidence for the claim shown in Fig. 1D, we designed a model experiment in that tag-purified topo IIβ was immobilized on magnetic beads and supercoiled plasmid DNA was used as a substrate. Flag-tagged topo IIβ was expressed in the human embryonal kidney cell line HEK293E cells transfected with pFlag-top2β plasmid encoding the full-length rat topo IIβ, and purified by immunoprecipitation with Dynabeads Protein G that had been pre-coated with anti-Flag antibody as described previously[2]. The plasmid harbored an insert originated from actual topo IIβ−target site[3], which is an AT-rich region containing a Ts2 toposite and LINE sequence (Fig. S2A). The enzyme acts on intramolecular crossovers in the circular substrate that are formed within insert or within vector portions.

The topo IIβ-bead (10 μl) was incubated with 100 ng of supercoiled substrate DNA in the presence or absence of 0.5 mM ATP/100 μM etoposide at 30°C for 30 min in 25 μl of reaction mixture containing the standard topo II buffer (50 mM Tris-HCl: pH 8.0, 120 mM KCl, 10 mM $MgCl_2$, 0.5 mM dithiothreitol, 0.5 mM EDTA, and 30 μg/ml BSA. All the reactions shown in panel B were terminated by addition of SDS/proteinase K (SDS/PK), and then applied to agarose gels. The ATP requirement for complete relaxation of form I to form Ir (relaxed closed circle) shows the absence of topoisomerase I contamination (Fig. S2B, lane 1). In the presence of etoposide, breakage products (forms II and III) were also formed in addition to relaxed form Ir (Fig. S2B, lane 3). The breakage products were shown to be bound on enzyme after magnetic separation (Fig. S2B, lane 4), whereas the relaxed form Ir stays in solution, indicating that the relaxed product is completely dissociated from the enzyme (not shown).

When the bound fraction (B) was incubated in the absence of etoposide with 1% sarkosyl-0.5 M CsCl (S/C) added in this order, form Ir newly emerges in B (Fig. S2C, lane 2). No DNA was detected in the unbound fraction (U) (Fig. S2C, lane 3). Absence of Ir in U is a clear contrast to the normal topo II reaction, in which Ir is released from the enzyme. This is a strong indication not only for residual activity of topo IIβ even in the presence of sarkosyl, but also for the enzyme's ability to bind DNA noncovalently under these conditions. The association of form Ir with the enzyme is quite stable and no Ir is released even with 0.5 M NaCl treatment (HS) despite the fact that the association is noncovalent in nature (Fig. S2C, lanes 4 and 5). As sarkosyl partially denatures the enzyme, the structure of topo II-DNA complex after S/C treatment should be altered to some extent. To see whether or not the altered complex binds nonspecific DNA, linearized pUC18 DNA (form III) was added in excess in the presence of S/C (Fig. S2C, lanes 6 and 7). Only a trace amount of the pUC18 DNA bound to the altered complex (Fig. S2C, lane 6), while the majority stayed in solution

(Fig. S2C, lane 7). HS treatment eluted the pUC18 DNA but not form Ir DNA (Fig. S2C, lane 9), suggesting that these DNAs reside in different compartments i.e. outside and inside the complex, respectively.

To test whether or not the circularity of form Ir DNA is required for its retention, DNA in the S/C-altered complex was treated with *Bam* HI *in situ* to separate into the insert and vector portions, followed by separation into bound and unbound fractions (Fig. S2D, lanes 3 and 4). The result showed that about half of the DNA stayed in B (lane 3). To see whether the bound fraction contained both covalently and noncovalently associated DNA, the B fraction was treated with 0.5 M NaCl (HS). The presence of DNA bands in U (Fig. S2D, lane 6) demonstrated that partially denatured enzyme does retain the noncovalently associated linear DNA. The reason why circular Ir was not released from the altered complex (Fig. S2C, lane 5) would be that their interaction derives from topological catenation as well as ionic association.

The agarose gel bands were quantified by densitometry (Fig. S2D, right panel). Comparison between lanes 1 and 2 suggested that the S/C treatment induced a breakage-resealing cascade in enzyme-bound DNAs: dsb (form III) to ssb (form II), and ssb to no break (form Ir). This is also a direct evidence for the preservation of religation activity and the dimeric structure of topo IIβ in the presence of sarkosyl.

Results are visualized schematically in Fig. S2E. Note that enzyme-bound dsb fragment is not detectable after *Bam* HI treatment because of their heterogeneous size and the bands in lane 5 are exclusively attributable to the ssb fragments. The insert to vector ratio in lanes 5 and 6 of Fig. S2E suggests that resealing efficiency of the enzyme bound to insert portion is significantly higher than that bound to vector portion. Crossovers between insert and vector portions are probably rare to occur because I/V ratio of high salt-released DNA should be unity in this case.

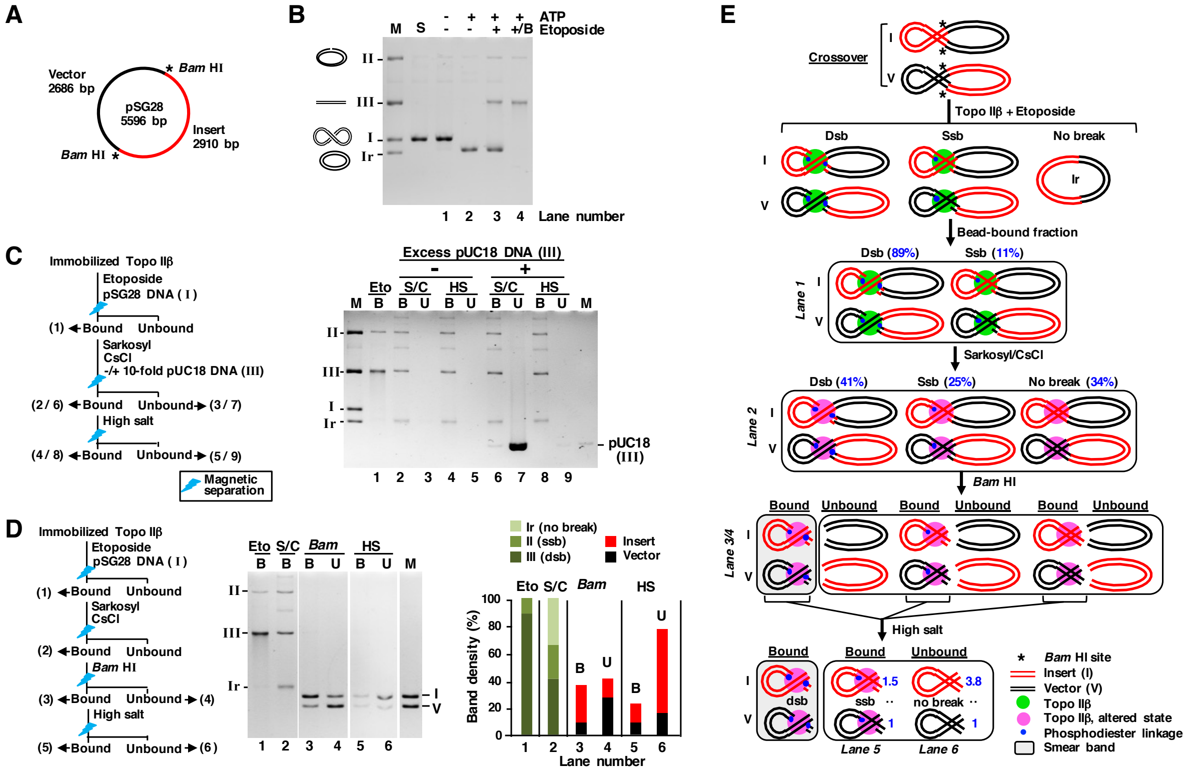

Supplementary Fig. S2 Reproduction of the eTIPa procedure in a model experiment using immobilized topo IIβ and plasmid DNA. (**A**) Circular map of the plasmid pSG28: sequence derived from a genomic region of topo IIβ target (chr1:63,270,479-63,273,382) was inserted into pUC18 vector. (**B**) Topo IIβ reaction with or without ATP/etoposide (indicated on top). Rat topo IIβ immobilized on magnetic beads via specific antibody was used throughout. After incubating the substrate DNA with topo IIβ-beads, the reaction was terminated by SDS and the whole products were analyzed by electrophoresis in agarose gels containing ethidium bromide, which can discriminate supercoiled (I), relaxed (Ir), nicked (II) and linear (III) DNA forms (illustrated on the left). M, marker mixture; S, substrate. The reaction applied to lane 3 was repeated and the bead-bound fraction (B) was isolated before adding SDS (lane 4). (**C**) Effects of sarkosyl/CsCl and addition of excessive DNA. Experimental procedure is outlined schematically on the left. Arrows indicate that samples were treated with SDS and proteinase K before loading onto agarose gels. Lane numbers are indicated in parentheses. B, bead-bound; U, bead-unbound. Eto, reaction with 100 μM etoposide; S/C, 1% sarkosyl+0.5 M CsCl; HS, 0.5 M NaCl (high salt). Reactions loaded on lanes 6-9 were the same as those loaded on lanes 2-5 except that 1 μg (10-fold excess of substrate) of linearized pUC18 was added before magnetic separation. Amount of unbound fraction loaded onto lane 7 was reduced to 1/10 of other Us to alleviate the probable interference to neighboring lanes. The rightmost lane M is a marker for form III pUC18. Lane 2 shows the resealing of etoposide-induced breakages during incubation with S/C. (**D**) Partial release of enzyme-bound

DNA after *in situ* digestion with a restriction enzyme. Experimental outline is shown on the left. The beads fraction shown on lane 2 was treated with *Bam* HI to cut the plasmid into insert (I) and vector (V) fragments followed by magnetic separation. Shown in lanes 3 and 4 are bead-bound DNA and released DNA, respectively. The bound DNA after *Bam* HI digestion (lane 3) was treated with 0.5 M NaCl to separate into bead-bound (lane 5) and unbound (lane 6) fractions. Enzyme-bound DNA fragments were partially released by HS treatment. The four portions of gel images separated by blank spaces were cropped from the same gel. The right panel: Densitometric analysis of the DNA bands separated by electrophoresis. Lanes in the central panel were scanned and band densities were quantified by the software associated with a gel imaging system. Peak areas in arbitrary unit were plotted as percentages in stacked bars. (**E**) Interpretation of the results illustrated schematically. Crossovers formed within the insert portion (labeled 'I') and within the vector portion (labeled 'V') are depicted pairwise. Three states of the G-segment cleavage are indicated (Dsb, Ssb, and No break). DNA fragments derived from dsb complex after restriction cutting are heterogeneous in length and are smeared out in the gel (shown in the shaded box). Numbers in blue indicate either percentage or ratio that are calculated from the densitometric values for the bands (specified by italicized lane numbers on left and bottom).

Based on the model experiment, now it would be safe to conclude: 1) The strand breakage induced by etoposide is partially resealed in the presence of detergent sarkosyl (Fig. S1A, step 2) as etoposide is significantly diluted-out once the cells were lysed. 2) The enzyme's resealing activity and association with the resealed DNA is preserved even in the presence of sarkosyl. Existence of Ts2 toposites in eTIPa-seq solely depends on the reversal of the gap on G-segments. T-segments may be also clamped to the partially denatured enzyme, probably after being transferred through the gap.

Critical factors in eTIP-seq procedure

In conventional immuno-capturing methods to separate the topo II-DNA cleaved complex, etoposide-treated cells are normally lysed by solutions containing SDS, a strong detergent, which denatures the enzyme instantly and dissociates all the DNA that are bound noncovalently. Thus, the fractionated topo II-DNA complex contains only DNA that is linked to the enzyme covalently (G-segment). In the present study, however, we lysed cells with a weaker detergent, sarkosyl (sodium lauroyl sarkosinate), to preserve the association of DNA interacting noncovalently with the enzyme, which may well include T-segments bound to the reaction intermediate. Although sarkosyl has been shown to dissociate histones effectively

from chromatin DNA[4], it leaves RNA polymerase II bound to template DNA without inactivating the enzyme, as revealed by continued transcription even in the presence of 1% sarkosyl in nuclear run-on assays[5,6]. Therefore, topo IIβ may also retain noncovalently bound DNA and a certain level of enzymatic activity in 1% sarkosyl. Since etoposide is diluted away after the cell lysis, the residual topo II activity may reseal the breaks in G-segment to some extent.

In the practical procedure, cell lysate in 1% sarkosyl was supplemented with 0.5 M CsCl to further remove materials interacting nonspecifically with topo IIβ. In the final step of eTIPa-seq, significant amounts of DNA are eluted in P2 fraction by 0.5 M NaCl treatment, implying that positively charged residues of topo IIβ interacting with phosphoryl groups of DNA are replaced by Na^+ ion but not by Cs^+ ion. This is consistent with the fact that the accumulation of alkali metal cations near the dsDNA phosphoryl groups varies inversely with its ionic size[7]. Thus, Na^+ is stronger than Cs^+ as an agent for eluting DNA fragments ionically bound to topo IIβ. The DNA yield in P2 is highly dependent on etoposide (Fig. S1D). This implies that these DNA fragments released by 0.5 M NaCl are not just irrelevant ones but are genuine components of topo IIβ reaction intermediates.

Etoposide-induced dsb mediated by topo IIβ might be repaired by microhomology-mediated end-joining (MMEJ) or by other homologous end-joining mechanisms. During this process, two distally located homologous sites would be somehow brought into proximity and processed into chimeric ligation products, namely DSP chimeras. However, this is unlikely to occur for number of reasons. i) Topo IIβ molecule should be removed from dsb ends before the start of repair end-joining process, which requires multiple protein factors. There should be little possibility that all these factors are associated with topo IIβ-DNA complex after cell lysis and can execute the expected reaction. ii) MMEJ usually operates between short homologous DNA segments in the vicinity of dsb site[8] that are not positioned kilobases apart from each other as in DSP chimeras. iii) The DSP chimera is derived from the ligation between two DNA fragments associated with topo IIβ. The ligation occurs between artificial adaptors attached to the ends of randomly sheared DNA. The adaptor serves as a signature for isolation of chimeras. iv) MMEJ-mediated ligation would never bring about the biased read orientations as observed in DSP chimeras (Fig. 4F).

2. Other genome-wide mapping for correlative analyses

We performed additional genome-wide analyses together with topo IIβ-targeted sites. Most attention was given to the binding sites of hnRNP U/SAF-A/SP120 as determined by ChIP-seq.

While any DNA, with a certain sequence preference, can be a substrate for topo II *in vitro*, accessibility of the enzyme to DNA *in vivo* is restricted significantly by bound chromatin proteins. Therefore, FAIRE-seq assays were employed to probe the local chromatin accessibility to evaluate potential topo IIβ targets.

These mapping results were displayed on custom tracks of the UCSC genome browser together with toposites. Using this display, a number of correlative analyses were performed as described in the text.

3. Supplementary results

Analysis of Ts1/PSP sites

Length distribution of Ts1/PSP showed that median length is around 700-800 bp (Fig. S3A). Location of Ts1/PSP chimera in the classified genomic regions was examined to assess their functional relevance (Fig. S3B). The remarkable enrichment of Ts1/PSP in TSS zone suggests that it is involved in the control of transcriptional initiation. We divided Ts1/PSP into two groups: associated and non-associated with TSS zone (Fig. S3C). Analysis of length distribution showed that TSS-associated Ts1/PSPs are significantly longer than the other group (Fig. S3D). With respect to the sequences around TSS (+/- 4 kb) extracted from rat RefSeq genes, Ts1/PSP and other features were plotted in an aggregation plot (Fig. S3E). Peak regions covering each genomic position were counted-up and expressed as relative numbers of RefSeq genes. SP120 and CpG island peak around TSS, whereas Ts1 toposite forms twin peaks that locate ~1,300 bp apart harboring the other peaks. These results are consistent with the model shown in Fig. S3F. As depicted in the figure, the left peak of Ts1 toposite very likely to correspond to the cleavage site of topo IIβ in action. The enzyme can also approach the duplex crossover from the other side, which should create the right peak. Taken together it is strongly suggested that topo IIβ complexed with SP120 recognizes a right-handed crossover formed between the positions of ~300 bp upstream TSS and ~1 kb downstream into the gene. In average genes, therefore, topo IIβ/SP120 acts on positively supercoiled loop of ~1,300 bp formed around TSS whose size matches well with the chimera size of ~1,200 bp (Fig. S3D). After strand passage, the loop is converted to negative loop, which may facilitate the initiation step of transcription[9]. Ts1/PSPs that are not associated with TSS but enriched in genic region may represent topo IIβ involved in the relaxation of positive supercoils generated by ongoing transcription[10]. In this case the loop also contains interaction sites for SP120 but the loop size is smaller (800-900 bp).

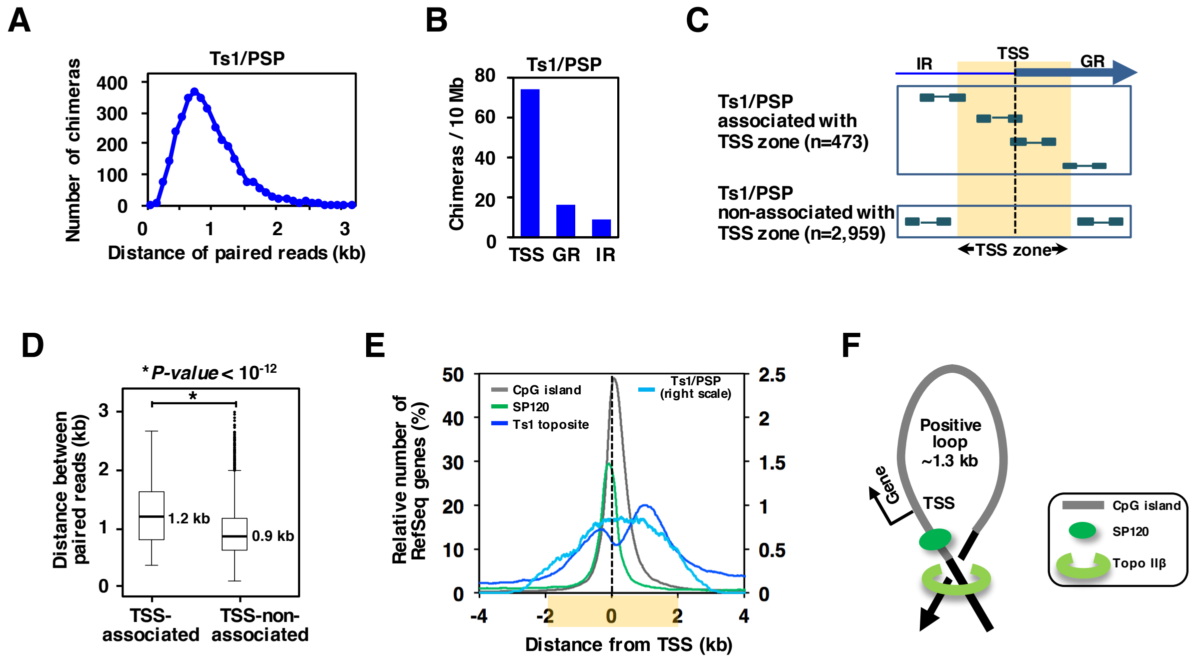

Supplementary Fig. S3 Analysis of Ts1/PSP chimeras. (**A**) Size distribution of Ts1/PSP chimera. (**B**) Location of Ts1/PSP chimeras in three genomic regions: TSS zone, genic region (GR), and intergenic region (IR), TSS zone being excluded from GR and IR. (**C**) Dividing Ts1/PSP into two groups on the basis of association with TSS zone (TSS +/- 2 kb). Sequence reads on chimera ends are designated by filled rectangles. (**D**) A box plot for length distribution of Ts1/PSP. Horizontal bars indicate median length. TSS-associated Ts1/PSP is significantly longer than non-associated ones. (**E**) Aggregation plot. CpG island, SP120 site, Ts1 toposite, and Ts1/PSP chimera overlapping with TSS zone (shadowed) were aggregated. Annotation data for RefSeq genes and CpG island were downloaded from the UCSC genome browser site. The right scale applies only to Ts1/PSP. (**F**) A model for the relationship between topo IIβ action site and SP120 binding site in the vicinity of TSS. This is based on the aggregation data shown in 'E'. A positive turn (PSP loop) is most likely to be formed in the region.

Analysis of DSP sites

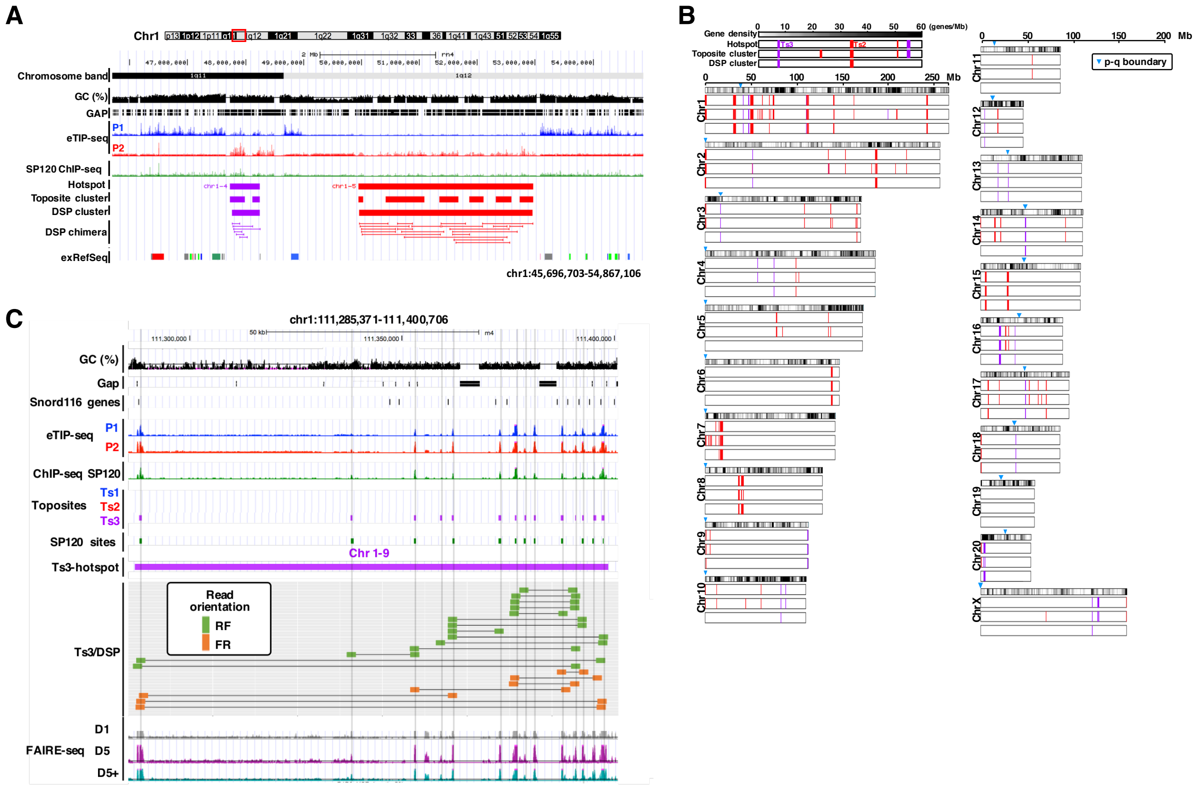

Supplementary Fig. S4 Analysis of DSP chimeras. **(A)** Positional relationships between the hotspots and toposite and DSP clusters. The exRefSeq on the bottom track represents a modified RefSeq genes categorized by expression patterns[11]. **(B)** Karyogram of hotspots with reference to gene density, toposite cluster, and DSP cluster. Gene density (number of genes per 1 Mb) is depicted by grayscale gradient. Tracks are arranged in the same order as in the legend shown at the top-left corner. Ts2 (red) and Ts3 (magenta) sites are shown by bars in the same track. The boundary between cytobands p and q marked by nabla symbol indicates approximate position of centromere. The karyogram plots were generated by R package ggbio[12]. (**C**) A browser view of the Snord 116 locus, a typical Ts3 hotspot. Entire region of the Ts3-hotspot (Chr1-9) is shown. Ts3 toposites are marked by vertical lines to indicate the accordance with the peaks in other tracks. FAIRE-seq assay was done at culture day 1 (D1), day 5 (D5), and day 5 in the presence of topo II inhibitor ICRF-193 (D5+). The Snord116 is a noncoding RNA gene (snoRNA), whose transcripts are involved in modification of rRNA and are expressed prevalently in the brain[13]. This locus overlaps with the Prader-Willi syndrome (PWS) region (corresponds to human chromosome 15q11-13), which is expressed only paternally and if deleted the subject may suffer developmental delay and mental retardation[14]. Read positions of all the Ts3/DSP chimeras mapped in this region coincided well with Ts3 toposites and SP120 sites. Their read orientations were either RF or FR. It is worth noting that

in accord with these sites high FAIRE-seq peaks were detected, indicating that these Ts3/DSP sites stay in an open chromatin conformation all the time in culture. The coincidence of these features is shared by all other Ts3-hotspots shown in Fig. S5.

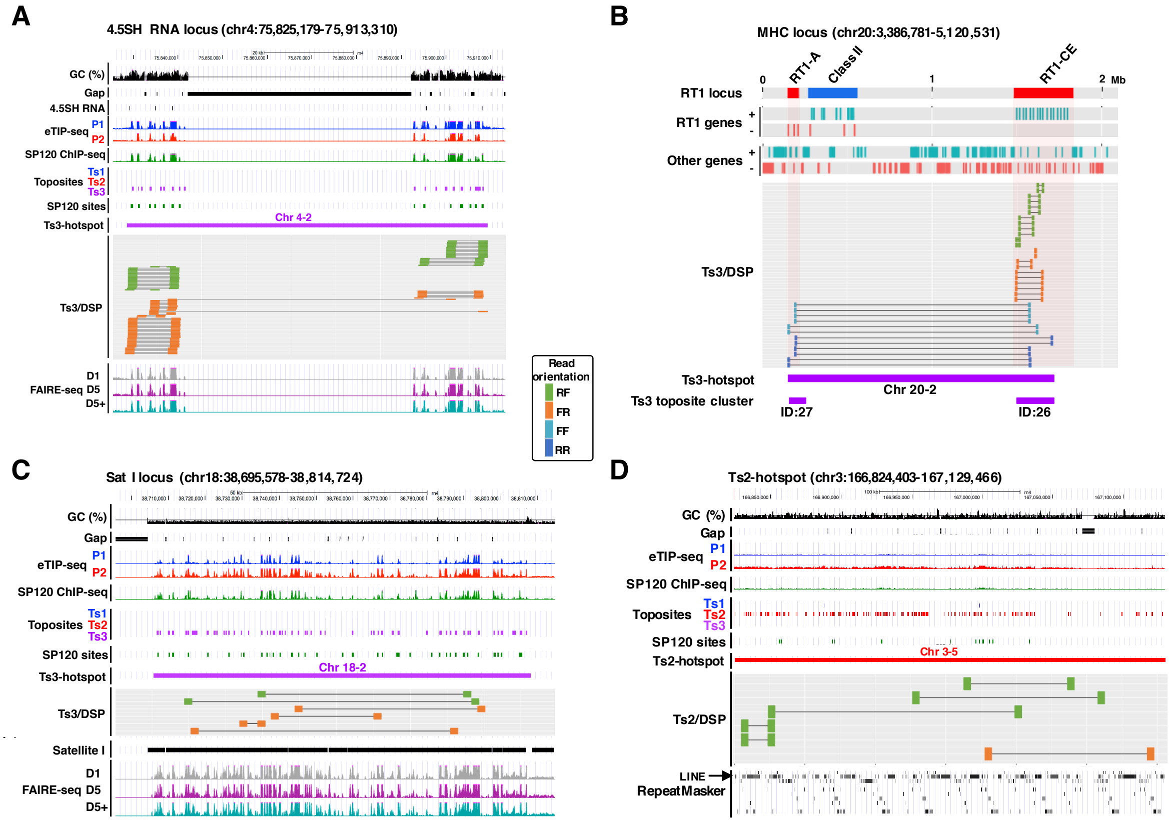

Supplementary Fig. S5 DSP hotspots that are worth featuring. (**A**) The Ts3-hotspot Chr4-2 corresponds to a noncoding RNA gene cluster (4.5SH RNA) which is unique to myomorpha, a large group in rodents including rats, mice, and hamsters. The rat 4.5SH RNA (~94 bp) is a part of a 5.3 kb unit that is transcribed by RNA pol III, several hundred copies of which are arranged as tandem repeats on this locus of rat chromosome 4[15]. In the rn4 browser view, the Ts3-hotspot is interrupted by a long sequence gap, whose position is very likely to be occupied by the same repeats flanking the gap and thus whole length may exceed 1 Mb. (**B**) The hotspot Chr20-2 is composed of two Ts3 toposite clusters (ID:26 and ID:27) and resides within the rat MHC gene cluster. Some Ts3/DSPs connecting these clusters are among the longest that exceed 1 Mb. Using the published complete sequence of rat MHC gene cluster[16], we improved the mapping accuracy of toposites and Ts3/DSPs on the reference sequence (rn4). As shown here (note that sequence direction is reversed), the rat MHC class Ia genes, termed RT1, are present in two gene clusters (designated RT1-A and RT1-CE). Reads belonging to Ts3/DSPs detected in this RT1 locus are all confined within the two Ts3 toposite clusters. In addition to intra-domain chimeras, cluster ID:26 contains long chimeras linking to

the neighboring cluster ID:27. Read orientations of intra-domain chimeras are all RF or FR, whereas inter-domain chimeras are all RR or FF. This must be related to the fact that, in rodents, the RT1-CE domain is partially duplicated and inserted inversely to generate the RT1-A domain[16], which is consistent with the fact that RT1 genes are coded by opposite strands in these domains. The connection between the read orientation and the coding strand observed here is an additional strong evidence for the rule shown in Fig. 4E. In contrast to RT1-A/CE genes, other RT1 genes and MHC class II genes are devoid of Ts3/DSP. (**C**) The hotspot Chr18-2 is a Ts3-hotspot that encompasses about 100 kb of pericentromeric region adjacent to the p-q boundary of the cytoband. All the Ts3/DSP reads in this region (either RF or FR orientation) coincide with the positions of Ts3 toposites, SP120 sites, FAIRE-seq peaks and Sat I sequences with high SW score from RepeatMasker. Sat I is a rat-specific repetitive element[17]. Multiple copies of Sat I repeats (unit length~370 bp) aligned in tandem constitute the centromeric region spanning megabases on most chromosomes in the rat[18]. Similar Sat I clusters were located in the pericentromeric region of Chr13, Chr17 and Chr18, as well. (**D**) The Ts2-hotspot Chr 3-5 encompassing about 300 kb is a typical hotspot enriched with Ts2 toposites and Ts2/DSP chimeras with RF/FR read orientation. The chimera ends overlap with LINE repeats in the RepeatMasker track.

Comparison to END-seq data

To investigate the relationship between the present study and other mapping techniques for topo IIβ-generated DSBs, we compared the peak positions of Ts1 toposite and those of DSB obtained by END-seq[19]. The END-seq data downloaded from NCBI SRA for mouse cortical neurons (END_seq_NRN_ETO) was first converted to fastq file and mapped to the mouse genome mm10 by Bowtie 1.1.2. Peaks of 57,961 were identified by a peak-calling algorithm (MACS 1.4.3). Using the LiftOver tool of UCSC genome browser, 26,560 peaks (46%) were successfully converted onto the rat genome rn4. Positions of these END-seq peaks were compared with those of 121,478 Ts1 toposites (listed in Supplementary Table S1). We found that 12,098 END-seq peaks (46%) were located within 500 bp from Ts1 toposites.

Analysis of gene expression by mRNA-seq

Total cellular RNA from D1, D5, and D5+ cells were prepared as described previously[11]. RNA-Sequencing libraries were generated using the Truseq SBS kit v3-HS (Illumina) with a polyA selection step. The samples were sequenced (75-bp paired-end sequencing) on one lane of HiSeq 2000 (Illumina) per sample. Expression levels of genes were determined based on FPKM values. Out of all exRefSeq-annotated genes (16,761 genes), 12,121 genes were

expressed at D5. In these expressed genes, 70.2% were not affected by ICRF-193 (0.66< D5/D5+ <1.5), whereas the remaining 29.8% were ICRF-affected. If we regard the genes with FPKM values higher than the 3rd quartile are highly expressed genes, 27.4% of ICRF-unaffected genes and 19.4% of affected genes were highly expressed, indicating that the expression level of ICRF-affected genes is not particularly high. Essentially the same results were obtained when FPKM values at D1 were used. Thus, these results suggest that in terminally differentiating neurons transcription may not be a major determinant in the alteration of chromatin compaction shown in Fig. 5. More complete results of expression analysis are described elsewhere[20].

References

1 Rashid, N. U., Giresi, P. G., Ibrahim, J. G., Sun, W. & Lieb, J. D. ZINBA integrates local covariates with DNA-seq data to identify broad and narrow regions of enrichment, even within amplified genomic regions. *Genome Biol* **12**, R67, doi:10.1186/gb-2011-12-7-r67 (2011).

2 Onoda, A. *et al.* Nuclear dynamics of topoisomerase IIβ reflects its catalytic activity that is regulated by binding of RNA to the C-terminal domain. *Nucleic Acids Res* **42**, 9005-9020, doi:10.1093/nar/gku640 (2014).

3 Miyaji, M., Furuta, R., Sano, K., Tsutsui, K. M. & Tsutsui, K. Genomic regions targeted by DNA topoisomerase IIβ frequently interact with a nuclear scaffold/matrix protein hnRNP U/SAF-A/SP120. *Journal of cellular biochemistry* **116**, 677-685, doi:10.1002/jcb.25024 (2015).

4 Scheer, U. Changes of nucleosome frequency in nucleolar and non-nucleolar chromatin as a function of transcription: an electron microscopic study. *Cell* **13**, 535-549, doi:10.1016/0092-8674(78)90327-6 (1978).

5 Fei, J. *et al.* NDF, a nucleosome-destabilizing factor that facilitates transcription through nucleosomes. *Genes Dev* **32**, 682-694, doi:10.1101/gad.313973.118 (2018).

6 Geen, M. H., Buss, J. & Gariglio, P. Activation of Nuclear RNA Polymerase by Sarkosyl. *Eur J Biochem* **53**, 217-225, doi:10.1111/j.1432-1033.1975.tb04060.x (1975).

7 Giambasu, G. M. *et al.* Competitive interaction of monovalent cations with DNA from 3D-RISM. *Nucleic Acids Res* **43**, 8405-8415, doi:10.1093/nar/gkv830 (2015).

8 Sallmyr, A. & Tomkinson, A. E. Repair of DNA double-strand breaks by mammalian alternative end-joining pathways. *J Biol Chem* **293**, 10536-10546, doi:10.1074/jbc.TM117.000375 (2018).

9 Corless, S. & Gilbert, N. Investigating DNA supercoiling in eukaryotic genomes. *Brief Funct Genomics* **16**, 379-389, doi:10.1093/bfgp/elx007 (2017).

10 Joshi, R. S., Pina, B. & Roca, J. Topoisomerase II is required for the production of long Pol II gene transcripts in yeast. *Nucleic Acids Res* **40**, 7907-7915, doi:10.1093/nar/gks626 (2012).

11 Sano, K., Miyaji-Yamaguchi, M., Tsutsui, K. M. & Tsutsui, K. Topoisomerase IIβ activates a subset of neuronal genes that are repressed in AT-rich genomic environment. *PLoS One* **3**, e4103, doi:10.1371/journal.pone.0004103 (2008).

12 Yin, T., Cook, D. & Lawrence, M. ggbio: an R package for extending the grammar of graphics for genomic data. *Genome Biol* **13**, R77 doi:https://doi.org/10.1186/gb-2012-13-8-r77 (2012).

13 Cavaille, J. *et al.* Identification of brain-specific and imprinted small nucleolar RNA genes exhibiting an unusual genomic organization. *Proc Natl Acad Sci U S A* **97**, 14311-14316, doi:10.1073/pnas.250426397 (2000).

14 Ohta, T. *et al.* Imprinting-mutation mechanisms in Prader-Willi syndrome. *Am J Hum Genet* **64**, 397-413, doi:10.1086/302233 (1999).

15 Gogolevskaya, I. K., Koval, A. P. & Kramerov, D. A. Evolutionary history of 4.5SH RNA. *Mol Biol Evol* **22**, 1546-1554, doi:10.1093/molbev/msi140 (2005).

16 Hurt, P. *et al.* The genomic sequence and comparative analysis of the rat major histocompatibility complex. *Genome Res* **14**, 631-639, doi:10.1101/gr.1987704 (2004).

17 Pech, M., Igo-Kemenes, T. & Zachau, H. G. Nucleotide sequence of a highly repetitive component of rat DNA. *Nucleic Acids Res* **7**, 417-432 (1979).

18 Takeiri, A. *et al.* New DNA probes to detect aneugenicity in rat bone marrow micronucleated cells by a pan-centromeric FISH analysis. *Mutat Res* **755**, 73-80, doi:10.1016/j.mrgentox.2013.05.011 (2013).

19 Canela, A. *et al.* Genome Organization Drives Chromosome Fragility. *Cell* **170**, 507-521 e518, doi:10.1016/j.cell.2017.06.034 (2017).

20 Miyaji, M. *et al.* Topoisomerase IIβ targets DNA crossovers formed between distant homologous sites to modulate chromatin structure and gene expression. *Preprint at bioRxiv*, https://doi.org/10.1101/484956 (2019).
